# Covalent Triazine Frameworks via a Low‐Temperature Polycondensation Approach

**DOI:** 10.1002/anie.201708548

**Published:** 2017-10-09

**Authors:** Kewei Wang, Li‐Ming Yang, Xi Wang, Liping Guo, Guang Cheng, Chun Zhang, Shangbin Jin, Bien Tan, Andrew Cooper

**Affiliations:** ^1^ Key Laboratory of Material Chemistry for Energy Conversion and Storage Ministry of Education School of Chemistry and Chemical Engineering, Huazhong University of Science and Technology Luoyu Road No. 1037 430074 Wuhan China; ^2^ Key Laboratory of Luminescence and Optical Information, Ministry of Education, School of Science Beijing Jiaotong University No.3 Shangyuancun, Haidian District 100044 Beijing China; ^3^ College of Life Science & Technology Huazhong University of Science and Technology Luoyu Road No. 1037 430074 Wuhan China; ^4^ Department of Chemistry and Materials Innovation Factory University of Liverpool Crown Street Liverpool L69 7ZD UK; ^5^ Tianjin Key Laboratory of Molecular Optoelectronic Sciences, Department of Chemistry Tianjin University, and Collaborative Innovation Center of Chemical Science and Engineering (Tianjin) Tianjin 300072 China

**Keywords:** covalent triazine frameworks, energy storage, gas adsorption, layered materials, photocatalysis

## Abstract

Covalent triazine frameworks (CTFs) are normally synthesized by ionothermal methods. The harsh synthetic conditions and associated limited structural diversity do not benefit for further development and practical large‐scale synthesis of CTFs. Herein we report a new strategy to construct CTFs (CTF‐HUSTs) via a polycondensation approach, which allows the synthesis of CTFs under mild conditions from a wide array of building blocks. Interestingly, these CTFs display a layered structure. The CTFs synthesized were also readily scaled up to gram quantities. The CTFs are potential candidates for separations, photocatalysis and for energy storage applications. In particular, CTF‐HUSTs are found to be promising photocatalysts for sacrificial photocatalytic hydrogen evolution with a maximum rate of 2647 μmol h^−1^ g^−1^ under visible light. We also applied a pyrolyzed form of CTF‐HUST‐4 as an anode material in a sodium‐ion battery achieving an excellent discharge capacity of 467 mAh g^−1^.

Covalent organic frameworks (COFs) are an emerging class of porous materials, characterized by their ordered structures, high surface areas, and structural diversity.^1^ They have shown promise in applications such as gas adsorption,^2^ catalysis,^3^ and optoelectronics.^4^ A variety of methods have been reported to prepare COFs, such as polycondensation,1a, 4a cyclization reactions,^5^ or surface mediated methods.1b, 6 Covalent triazine frameworks (CTFs) are related to COFs and are typically constructed through cyclization reaction of nitrile aromatic building blocks; they feature high physicochemical stability and high nitrogen content.^5^, ^7^ Because of these characteristics, CTFs have found diverse applications in gas adsorption and storage,5a, 7a,7b catalysis,7c–7e and energy storage.7f,7g There are still, however, a limited number of approaches for the synthesis of CTFs.5a, 7a The most common approach is ionothermal synthesis at high temperatures (≥400 °C), which also requires a large amount of ZnCl_2_ to serve as both catalyst and reaction medium.5a This method can lead to CTFs with a degree of crystalline order, but the high reaction temperatures cause the partial carbonization of the structure and the materials are obtained in the form of black powders. Hence, CTFs prepared by this method lack an electronic band gap and may be unsuitable for photophysical applications. Furthermore, these reaction temperatures consume a large amount of energy and preclude all but the most stable building blocks, thus limiting the scope for scale up and synthetic diversity. It is, therefore, imperative to find new methods for the synthesis of CTFs under milder conditions. Previous research has shown that CTFs could be synthesized at room temperature, and catalyzed by strong and corrosive acid such as trifluoromethylsufonic acid.7a,7b This avoids carbonization, but the method is obviously not suitable to acid‐sensitive building blocks, and also the resulting materials did not have layered structures.

Here, we develop a new strategy involving the condensation reaction of aldehydes and amidines to construct CTFs under mild conditions (Scheme 1). The newly‐synthesized CTFs possess layered structures, high surface areas, and tunable functions and geometries. An important feature of this method lies in the relatively gentle reaction conditions (≤120 °C, no strong acids). This general method enables CTFs to be easily scaled up to multigram level with no special apparatus. The materials are generally yellow or orange powders, which retain their optical band gap, suggesting potential applications in photophysical applications.^8^ Intriguingly, the CTFs display a layered structure and opening up a route to new type of nitrogen‐doped layered materials. These CTFs could be promising materials for the applications in photocatalaysis and energy storage, as exemplified here for photocatalytic sacrificial hydrogen evolution and in the formation of a sodium‐ion battery anode.

**Scheme 1:**
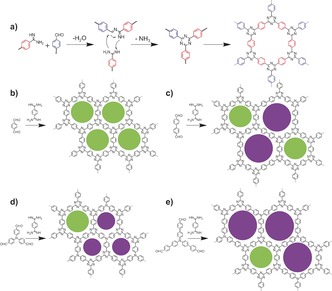
A Scheme showing reaction mechanism for CTF‐HUST synthesis. a) Reaction mechanism for triazine formation in the synthesis of CTF‐HUST; representative structures of b) CTF‐HUST‐1, c) CTF‐HUST‐2, d) CTF‐HUST‐3, and e) CTF‐HUST‐4; the circles filled with different colors represent the presence of two types of pores. “HUST” is the abbreviation of “Huazhong University of Science and Technology”.

The CTF materials were synthesized via a condensation reaction between an aldehyde and an amidine dihydrochloride involving a Schiff base formation followed by a Michael addition.^9^ This is the first time that this type of condensation reaction has been used for the synthesis of porous organic materials. The CTFs prepared from four aldehydes, 1,4‐benzene‐dialdehye, 4,4′‐biphenyl‐dialdehyde, tris(4‐formylphenyl)‐amine, and tris(4‐formylbiphenyl)‐amine, were named as CTF‐HUST‐1, CTF‐HUST‐2, CTF‐HUST‐3 and CTF‐HUST‐4, respectively (Scheme 1). CTF‐HUST‐1 was used as a model to optimize the reaction conditions. After screening different solvents, the optimal solvent was found to be dimethyl sulfoxide (DMSO) (Table S1 in the Supporting Information). The optimal reaction conditions involved the use of cesium carbonate (Cs_2_CO_3_) as a base, DMSO as a solvent, and a reaction temperature of 120 °C. As a comparison, solvothermal methods involving a closed reactor, as often used in the COFs syntheses, were also used for the CTF‐HUST‐1 preparation but no significant improvement in yield or properties was observed. Scalability is a key requirement for practical applications of new materials. We believe that this process could easily be scaled up because it is a one‐pot polymerization carried out at relatively low temperature and ambient pressure, without protection by an inert atmosphere, using a cheap catalyst and a relatively acceptable solvent. We successfully scaled up the polymerization of CTF‐HUST‐1 to 5 grams scale, without the use of any special equipment (Figure S1), and we believe that much larger scale syntheses should also be possible.

The successful formation of the triazine fameworks was confirmed by Fourier‐transformed infrared (FT‐IR) analysis, in which the characteristic vibrations from the triazine units at 1523 and 1367 cm^−1^ were observed for all CTF‐HUSTs (Figure S2), in good agreement with those of a small‐molecule model compound (Figure S2). The solid‐state cross‐polarization magic angle spinning carbon‐13 nuclear magnetic resonance (CP‐MAS ^13^C‐NMR) also unambiguously confirmed the formation of triazine structures in CTF‐HUSTs, where the chemical shifts at 122.0 and 132.4 ppm can be assigned to phenyl carbons and the chemical shift at 165.4 ppm is assigned to the carbon signal from triazine rings (Figure S3). Particle‐like morphologies were observed by field‐emission scanning electron microscopy (FE‐SEM) (Figure S4). The CTF‐HUSTs also show high thermal stability and are stable up to 550 °C without significant loss of mass, as revealed by thermal gravimetric analysis (TGA) measurements (Figure S5). Elemental analysis shows that the carbon and nitrogen contents are close to theoretical results (Table S2).

To investigate whether these framework materials have any long range ordering, we used powder X‐ray diffraction (PXRD). Despite the extensive optimization efforts, no crystalline CTFs were obtained. While these CTFs do not exhibit long‐range crystalline order (Figure 1 a and Figure S6, S7), PXRD data for CTF‐HUST‐1 showed two broad peaks at 7.6° and 25.8° (Figure 1 a, blue curve). CTF‐HUST‐2 exhibited two features at approximately 6° and 25° (Figure S7a, blue curve); CTF‐HUST‐3 contained two features at approximately 8° and 22° (Figure S7d, blue curve), and CTF‐HUST‐4 showed two features at around 6° and 21° (Figure S7g, blue curve). These features are very broad, even with respect to the relatively broad peaks observed for CTFs produced by ionothermal routes,7b and it is not possible to match these to a specific structural model. These features do suggest, however, the possibility of at least partially layered structures.

**Figure 1:**
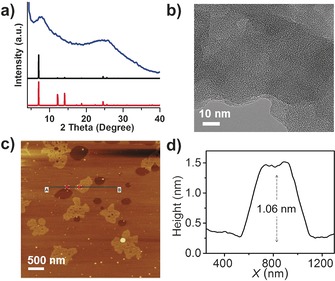
a) PXRD pattern of experimental (blue), simulated AA stacking (black) and simulated AB stacking (red) of CTF‐HUST‐1. b) High‐Resolution TEM image of CTF‐HUST‐1. c) Atomic force microscopy topography of CTF‐HUST‐1. d) Height profile of AFM of the corresponding edge height of CTF‐HUST‐1.

We further characterized the materials by high‐resolution transmission electron microscopy (HR‐TEM) and atomic force microscopy (AFM) as shown in Figure 1 b–d and Figure S8. HR‐TEM images are consistent with extended porous network structures. By carefully observing the HR‐TEM images, we noticed that the CTFs seem to show stacking or layer structures on their particle edges (Figure 1 b and Figure S8a–c). We further confirmed the layer structures in these CTFs by AFM imaging. We found that these CTFs can indeed exist as sheets that seem to be just a layers thick, when prepared by solvent‐assisted sonication in solvents such as ethanol. These CTF‐HUSTs could be well dispersed into ethanol, with concentration to be about 0.1 mg mL^−1^ (Figure S9) and AFM characterization was performed by first dispersing the samples in ethanol (Figure 1 c and Figure S8d–f). For instance, CTF‐HUST‐1 in ethanol was observed to have lamellar layers with thickness of 1.06 nm, whereas CTF‐HUST‐2, CTF‐HUST‐3 and CTF‐HUST‐4 give lamellar layers with thickness of 1.15 nm, 1.01 nm and 0.80 nm, respectively (Figure 1 b and Figure S8g–i). Some multilayered structures could also be observed by AFM (Figure S10). For instance, multilayered CTF‐HUST‐1 that are observed to bear an average layer thickness to be around 3.26 nm. Therefore, these results showed that the present CTF‐HSUTs are layered materials, or at least that a dispersible proportion of the sample is thus structured.

We next investigated the porosity of the CTF‐HUSTs. First, the materials were examined by nitrogen sorption experiments at 77 K. The surface areas and pore dimensions were evaluated based on Brunauer–Emmett–Teller (BET) and Langmuir methods, respectively. As shown in Figure 2 a, the nitrogen adsorption isotherms of the CTF‐HUSTs exhibit a steep rise at low relative pressure (*P*/*P*_0_<0.001), indicating a microporous structure. For CTF‐HUST‐2, the additional steep rise at high pressure (*P*/*P*_0_≈1) may be due to condensation in macropores formed between the highly aggregated particles.^10^ Nitrogen adsorption and desorption isotherms for CTF‐HUST‐4 show a clear hysteresis loop, which indicates the presence of mesopores. The calculated surface areas and pore volumes are summarized in Table S4. The pore size distributions of CTF‐HUSTs were calculated by using a nonlocal density functional theory (DFT) method (Figure 2 b). A pore size of around 12 Å was observed for all the CTFs, in agreement with theoretical values calculated from the idealized, crystalline models for the four CTF‐HUSTs (Scheme 1, green circles). The second type of pore apertures suggested by simulations for CTF‐HUST‐2, CTF‐HUST‐3 and CTF‐HUST‐4 (Scheme 1 c–e) were also observed by experiment (see shoulder peaks in Figure 2 b), although these materials are only partially ordered, and a distribution of pore sizes is often observed in purely amorphous porous polymers.7d Similarly, such peaks can be artifacts of the NL‐DFT analysis. We further investigated the carbon dioxide and hydrogen adsorption properties of these CTF‐HUSTs (Figure 2 c–d and Figure S11). CTF‐HUST‐2 has the highest hydrogen uptake in this series (1.28 wt % at 1.00 bar and 77 K, Figure 2 c) and CTF‐HUST‐3 has the highest CO_2_ uptake (13.91 wt % at 273 K, Figure 2 d). The isosteric heat of adsorption was also calculated from the CO_2_ isotherms measured at 273 K and 298 K (Figure S11b). At the onset of adsorption, the heat of adsorption of CO_2_ was 30, 29, 33 and 32 kJ mol^−1^ for CTF‐HUST‐1, CTF‐HUST‐2, CTF‐HUST‐3, and CTF‐HUST‐4, respectively (Figure S11b), which is in a good range for CO_2_ capture applications and higher than reported for CTF‐1 (27.5 kJ mol^−1^).^11^ Indeed, the CO_2_ adsorption heat for these CTF‐HUSTs is higher than for most other nitrogen‐rich porous organic frameworks, including porous benzimidazole polymers,^12^ electron‐rich organonitridic frameworks,^13^ conjugated microporous polymers,^14^ and hypercross‐linked polymers.^15^

**Figure 2:**
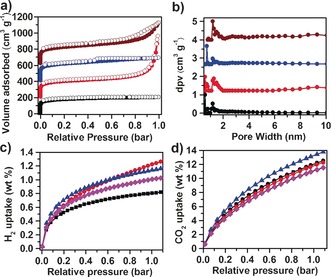
a) Nitrogen sorption curves and b) corresponding pore size distributions of CTF‐HUST‐1(black curve), CTF‐HUST‐2 (red curve), CTF‐HUST‐3 (blue curve) and CTF‐HUST‐4 (wine curve). c) H_2_ sorption curves of CTF‐HUST‐1 (black curve), CTF‐HUST‐2 (red curve), CTF‐HUST‐3 (blue curve) and CTF‐HUST‐4 (magenta curve). d) CO_2_ sorption curves of CTF‐HUST‐1(black curve), CTF‐HUST‐2 (red curve), CTF‐HUST‐3 (blue curve) and CTF‐HUST‐4 (magenta curve) at 273 K.

We also studied the optical properties of these CTF‐HUST samples and found that these materials could absorb light in the visible region up to around 650 nm (Figure 3 a–e). The band gaps were calculated for these CTF‐HUSTs on the basis of their UV‐visible absorption spectra. It was found that CTF‐HUST‐4 has the smallest band gap of 2.13 eV and CTF‐HUST‐2 has the largest gap (2.42 eV). The band gaps of CTF‐HUST‐1 and CTF‐HUST‐3 are 2.33 eV and 2.22 eV, respectively. The optical photographs of solid state CTF‐HUSTs are shown in Figure 3 b–e, showing that the colors of the samples are in line with their absorption properties. The different optical band gaps in this series indicate that the band gaps are tunable by varying the building blocks.8c

**Figure 3:**
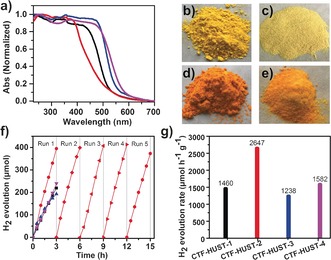
a) UV/Visible spectra of CTF‐HUST‐1 (black), CTF‐HUST‐2(red), CTF‐HUST‐3(blue) and CTF‐HUST‐4(magenta) in the solid state. Solid state image of b) CTF‐HUST‐1, c) CTF‐HUST‐2, d) CTF‐HUST‐3 and e) CTF‐HUST‐4. f) Time course of H_2_ evolution of CTF‐HUST‐1(black curve), CTF‐HUST‐2 (red curve), CTF‐HUST‐3 (blue curve) and CTF‐HUST‐4 (magenta curve) (>420 nm) and stability test for CTF‐HUST‐2 for running over 5 times. g) H_2_ evolution rate of CTF‐HUSTs.

The CTFs synthesized by conventional methods have been explored in photocatalysis, such as photocatalytic water splitting, because of their analogous structures to graphitic carbon nitrides.8a, 16 However, the photocatalytic activities are far from satisfactory. Combining layer structures with above‐mentioned optical properties of CTF‐HUSTs (tunable band gaps and broad visible absorption, Figure 3 a), CTF‐HUSTs are particular interesting for photocatalysis applications. We thereby tested our newly synthesized CTF‐HUSTs in sacrificial photocatalytic water splitting under visible light (>420 nm, Figure 3 f). CTF‐HUST‐1 showed a hydrogen evolution rate as 1460 μmol h^−1^ g^−1^, which is higher than reported for CTF‐1 synthesized by a modified ionothermal method (1072 μmol h^−1^ g^−1^).16a CTF‐HUST‐2 showed the highest photocatalytic hydrogen evolution rate in this series of 2647 μmol h^−1^ g^−1^. CTF‐HUST‐3 and CTF‐HUST‐4 exhibited hydrogen evolution rates of 1238 μmol h^−1^ g^−1^ and 1582 μmol h^−1^ g^−1^, respectively. These values are among the highest reported for porous organic materials. To our knowledge, the photocatalytic activity of CTF‐HUST‐2 is the highest reported for CTFs to date and higher than reported for COF materials with more crystalline structures,4e,4g although it must be stated here that these rates depend strongly on the specific set up used for the photolysis. Nonetheless, with that caveat, the hydrogen evolution of CTF‐HUST‐2 is higher than amorphous CTFS_10_ (2000 μmol h^−1^ g^−1^),16c a crystalline hydrazone‐based COF, TFPT‐COF (1970 μmol h^−1^ g^−1^)4e and azine‐based N_3_‐COF (1703 μmol h^−1^ g^−1^).4g The H_2_ production rates of theses CTF‐HUST materials are also comparable with graphitic carbon nitrides, such as *mpg*‐C_3_N_4_ (1490 μmol h^−1^ g^−1^) and crystalline PTI nanosheets (1750 μmol h^−1^ g^−1^).16c,16d The high photocatalytic activity could be attributed in part to the layered structures, which may be beneficial for photocatalysis because the thin layers could shorten the migration of generated charges and suppress the charge recombination. The stability of the CTF‐HUST‐2 photocatalyst was also studied. Photocatalytic hydrogen evolution could be observed for CTF‐HUST‐2 more than 5 times without any obvious loss of activity over a total of 15 h irradiation time (Figure 3 f).

Besides potential applications in photocatalysis, layered materials have also been explored extensively for energy storage, for example carbonaceous layered materials have been studied as anode materials for lithium‐ion battery (LIBs) or sodium‐ion battery (SIBs).^17^ Sodium‐ion battery is a promising alternative to lithium‐ion battery because sodium has lower cost and is more abundant. However, because sodium ion has a larger space than lithium, the interlayer distance is important for an effective electrode materials in SIBs. For example, graphite has been commercially available as Li‐ion batteries, however, it cannot work as efficient SIB anode materials because of the small interlayer distance that impedes the insertion and extraction of sodium ions.^18^ Recently, a COF‐derived N‐doped carbonaceous material was reported to work as efficient anode materials in SIBs.^19^ Inspired by the layer structures, we performed the pyrolysis of the CTF‐HUSTs (*p*‐CTF‐HUSTs) and characterized the resulting materials (Figure S12). *p*‐CTF‐HUST‐4 has a largest interlayer distance of 3.9 Å in the series (Figure S12d). It is known that a minimum 3.7 Å interlayer distance is required for an effective sodium transportation for SIBs.^18^ Therefore, *p*‐CTF‐HUST‐4 is promising candidates for SIBs. We further observed the AFM images of these pyrolyzed CTFs and found that *p*‐CTF‐HUSTs also have layered structures after pyrolysis, which may be either due to the preexisting layered structures or subsequent graphitization of the materials during pyrolysis (Figure S13). As a proof of concept, we thereby tested *p*‐CTF‐HUST‐4 as an anode material for SIBs. It was found that *p*‐CTF‐HUST‐4 exhibited high reversible capacity and excellent cycling and rate performances. A relatively small irreversible capacity was observed within the voltage range 0.05–2.0 V (vs. Na/Na^+^). As shown in Figure S14a, the initial discharge capacity was 467 mAh g^−1^, which compares favorably with lithium‐ion battery performance,^20^ although many other factors such as volumetric capacity, potential of reaction, and charge/discharge rates are also important for real devices. The material also shows high coulombic efficiency, rate performance and stability (Figure S14b–d).

In summary, we have developed a new strategy to construct covalent triazine frameworks by a novel polycondensation reaction under mild, potentially scalable conditions, avoiding high reactions temperatures or strong acids. Four layered CTF‐HUST materials with different building blocks and geometries were prepared. The CTF‐HUSTs displayed good performance in gas adsorption, photocatalysis and, after pyrolysis, in sodium‐ion battery applications. These findings provide a novel strategy for the design and synthesis of new classes of robust, functional earth‐abundant CTF materials for a variety of processes in energy storage, energy production and separation.

## Conflict of interest

The authors declare no conflict of interest.

## Supporting information

As a service to our authors and readers, this journal provides supporting information supplied by the authors. Such materials are peer reviewed and may be re‐organized for online delivery, but are not copy‐edited or typeset. Technical support issues arising from supporting information (other than missing files) should be addressed to the authors.

Supplementary

## References

[1a] A. P. Côté , A. I. Benin , N. W. Ockwig , M. O'Keeffe , A. J. Matzger , O. M. Yaghi , Science 2005, 310, 1166–1170; 16293756 10.1126/science.1120411

[1b] J. W. Colson , A. R. Woll , A. Mukherjee , M. P. Levendorf , E. L. Splitler , V. B. Shields , M. G. Spencer , J. Park , W. R. Dichtel , Science 2011, 332, 228–231; 21474758 10.1126/science.1202747

[1c] X. Feng , X. Ding , D. Jiang , Chem. Soc. Rev. 2012, 41, 6010–6022; 22821129 10.1039/c2cs35157a

[1d] S.-Y. Ding , W. Wang , Chem. Soc. Rev. 2013, 42, 548–568; 23060270 10.1039/c2cs35072f

[1e] B. P. Biswal , S. Chandra , S. Kandambeth , B. Lukose , T. Heine , R. Banerjee , J. Am. Chem. Soc. 2013, 135, 5328–5331; 23521070 10.1021/ja4017842

[1f] Q. Fang , Z. Fang , S. Gu , R. B. Kaspar , J. Zheng , J. Wang , S. Qiu , Y. Yan , Nat. Commun. 2014, 5, 1; 10.1038/ncomms5503 25054211

[1g] T.-Y. Zhou , S.-Q. Xu , Q. Wen , Z.-F. Pang , X. Zhao , J. Am. Chem. Soc. 2014, 136, 15885–15888; 25360771 10.1021/ja5092936

[1h] P. J. Waller , F. Gándara , O. M. Yaghi , Acc. Chem. Res. 2015, 48, 3053–3063; 26580002 10.1021/acs.accounts.5b00369

[1i] G. Lin , H. Ding , D. Yuan , B. Wang , C. Wang , J. Am. Chem. Soc. 2016, 138, 3302–3305. 26926489 10.1021/jacs.6b00652

[2a] H. Furukawa , O. M. Yaghi , J. Am. Chem. Soc. 2009, 131, 8875–8883; 19496589 10.1021/ja9015765

[2b] C. J. Doonan , D. J. Tranchemontagne , T. G. Glover , J. R. Hunt , O. M. Yaghi , Nat. Chem. 2010, 2, 235–238. 21124483 10.1038/nchem.548

[3a] S.-Y. Ding , J. Gao , Q. Wang , Y. Zhang , W. G. Song , C.-Y. Su , W. Wang , J. Am. Chem. Soc. 2011, 133, 19816–19822; 22026454 10.1021/ja206846p

[3b] A. Nagai , X. Chen , X. Feng , X. Ding , Z. Guo , D. Jiang , Angew. Chem. Int. Ed. 2013, 52, 3770–3774; 10.1002/anie.201300256 23436400

[3c] P. Pachfule , S. Kandambeth , D. D. Díaz , R. Banerjee , Chem. Commun. 2014, 50, 3169–3172. 10.1039/c3cc49176e 24519675

[4a] E. L. Spitler , W. R. Dichtel , Nat. Chem. 2010, 2, 672–677; 20651731 10.1038/nchem.695

[4b] E. L. Spitler , J. W. Colson , F. J. Uribe-Romo , A. R. Woll , M. R. Giovino , A. Saldivar , W. R. Dichtel , Angew. Chem. Int. Ed. 2012, 51, 2623–2627; 10.1002/anie.201107070 22223402

[4c] X. Ding , L. Chen , Y. Honsho , X. Feng , O. Saengsawang , J. Guo , A. Saeki , S. Seki , S. Irle , S. Nagase , V. Parasuk , D. Jiang , J. Am. Chem. Soc. 2011, 133, 14510–14513; 21863859 10.1021/ja2052396

[4d] M. Dogru , M. Handloser , F. Auras , T. Kunz , D. Medina , A. Hartschuh , P. Knochel , T. Bein , Angew. Chem. Int. Ed. 2013, 52, 2920–2924; 10.1002/anie.201208514 23382014

[4e] L. Stegbauer , K. Schwinghammer , B. V. Lotsch , Chem. Sci. 2014, 5, 2789–2793;

[4f] S. Lin , C. S. Diercks , Y. B. Zhang , N. Kornienkol , E. M. Nichols , Y. Zhao , A. R. Paris , D. Kim , P. Yang , O. M. Yaghi , C. J. Chang , Science 2015, 349, 1208–1213; 26292706 10.1126/science.aac8343

[4g] V. S. Vyas , F. Haase , L. Stegbauer , G. Savasci , F. Podjaski , C. Ochsenfeld , B. V. Lotsch , Nat. Commun. 2015, 6, 8508. 26419805 10.1038/ncomms9508 PMC4598847

[5a] P. Kuhn , M. Antonietti , A. Thomas , Angew. Chem. Int. Ed. 2008, 47, 3450–3453; 10.1002/anie.200705710 18330878

[5b] M. J. Bojdys , J. Jeromenok , A. Thomas , M. Antonietti , Adv. Mater. 2010, 22, 2202–2205. 20564256 10.1002/adma.200903436

[6] N. A. Zwaneveld , R. Pawlak , M. Abel , D. Catalin , D. Gigmmes , B. Denis , L. Porte , J. Am. Chem. Soc. 2008, 130, 6678–6679. 18444643 10.1021/ja800906f

[7a] S. Ren , M. J. Bojdys , R. Dawson , A. Laybourn , Y. Z. Khimyak , D. J. Adams , A. I. Cooper , Adv. Mater. 2012, 24, 2357–2361; 22488602 10.1002/adma.201200751

[7b] X. Zhu , C. Tian , S. M. Mahurin , S.-H. Chai , C. Wang , S. Brown , G. M. Veith , H. Luo , H. Liu , S. Dai , J. Am. Chem. Soc. 2012, 134, 10478–10484; 22631446 10.1021/ja304879c

[7c] C. E. Chan-Thaw , A. Villa , P. Katekomol , D. Su , A. Thomas , L. Prati , Nano Lett. 2010, 10, 537–541; 20085344 10.1021/nl904082k

[7d] L. Hao , J. Ning , B. Luo , B. Wang , Y. Zhang , Z. Tang , J. Yang , A. Thomas , L. Zhi , J. Am. Chem. Soc. 2015, 137, 219–225; 25496249 10.1021/ja508693y

[7e] R. Palkovits , M. Antonietti , P. Kuhn , A. Thomas , F. Schüth , Angew. Chem. Int. Ed. 2009, 48, 6909–6912; 10.1002/anie.200902009 19655358

[7f] H. Liao , H. Ding , B. Li , X. Ai , C. Wang , J. Mater. Chem. A 2014, 2, 8854–8858;

[7g] K. Schwinghammer , S. Hug , M. B. Mesch , J. Senker , B. V. Lotsch , Energy Environ. Sci. 2015, 8, 3345–3353.

[8a] R. S. Sprick , J.-X. Jiang , B. Bonillo , S. Ren , T. Ratvijitvech , P. Guiglion , M. A. Zwijnenburg , D. J. Adams , A. I. Cooper , J. Am. Chem. Soc. 2015, 137, 3265–3270; 25643993 10.1021/ja511552k

[8b] B. Bonillo , R. S. Sprick , A. I. Cooper , Chem. Mater. 2016, 28, 3469–3480;

[8c] R. S. Sprick , B. Bonillo , M. Sachs , R. Clowes , J. R. Durrant , D. J. Adams , A. I. Cooper , Chem. Commun. 2016, 52, 10008–10011. 10.1039/c6cc03536a 27443392

[9] S. Biswas , S. Batra , Eur. J. Org. Chem. 2012, 3492–3499.

[10] K. S. W. Sing , D. H. Everett , R. A. W. Haul , L. Moscou , R. A. Pierotti , J. Rouquérol , T. Siemieniewska , Pure Appl. Chem. 1985, 57, 603–619.

[11] Y. Zhao , K. X. Yao , B. Teng , T. Zhang , Y. Han , Energy Environ. Sci. 2013, 6, 3684–3692.

[12a] M. G. Rabbani , H. M. El-Kaderi , Chem. Mater. 2011, 23, 1650–1653;

[12b] M. G. Rabbani , T. E. Reich , R. M. Kassab , K. T. Jackson , H. M. El-Kaderi , Chem. Commun. 2012, 48, 1141–1143; 10.1039/c2cc16986j 22167176

[12c] M. G. Rabbani , H. M. El-Kaderi , Chem. Mater. 2012, 24, 1511–1517.

[13] P. Mohanty , L. D. Kull , K. Landskron , Nat. Commun. 2011, 2, 401. 21772272 10.1038/ncomms1405

[14] R. Dawson , D. J. Adams , A. I. Cooper , Chem. Sci. 2011, 2, 1173–1177.

[15] Y. Luo , B. Li , W. Wang , K. Wu , B. Tan , Adv. Mater. 2012, 24, 5703–5707. 23008146 10.1002/adma.201202447

[16a] S. Kuecken , A. Acharjya , L. J. Zhi , M. Schwarze , R. Schomacker , A. Thomas , Chem. Commun. 2017, 53, 5854–5857; 10.1039/c7cc01827d 28504790

[16b] J. H. Bi , W. Fang , L. Y. Li , J. Y. Wang , S. J. Liang , Y. H. He , M. H. Liu , L. Wu , Macromol. Rapid Commun. 2015, 36, 1799–1805; 26292975 10.1002/marc.201500270

[16c] L. Y. Li , W. Fang , P. Zhang , J. H. Bi , Y. H. He , J. Y. Wang , W. Y. Su , J. Mater. Chem. A 2016, 4, 12402–12406;

[16d] X. Wang , K. Maeda , X. Chen , K. Takanabe , K. Domen , Y. Hou , X. Fu , M. Antonietti , J. Am. Chem. Soc. 2009, 131, 1680; 19191697 10.1021/ja809307s

[16e] K. Schwinghammer , M. B. Mesch , V. Duppel , C. Ziegler , J. Senker , B. V. Lotsch , J. Am. Chem. Soc. 2014, 136, 1730; 24432762 10.1021/ja411321s

[16f] R. S. Sprick , B. Bonillo , R. Clowes , P. Guiglion , N. J. Brownbill , B. J. Slater , F. Blanc , M. A. Zwijnenburg , D. J. Adams , A. I. Cooper , Angew. Chem. Int. Ed. 2016, 55, 1792; 10.1002/anie.201510542 PMC4755226 26696450

[17a] A. C. Ferrari , Solid State Commun. 2007, 143, 47–57;

[17b] Y. Dall'Agnese , P. L. Taberna , Y. Gogotsi , P. Simon , J. Phys. Chem. Lett. 2015, 6, 2305–2309; 26266609 10.1021/acs.jpclett.5b00868

[17c] Z. G. Luo , J. Zhou , L. R. Wang , G. Z. Fang , A. Q. Pan , S. Q. Liang , J. Mater. Chem. A 2016, 4, 15302–15308;

[17d] L. L. Peng , Y. Zhu , D. H. Chen , R. S. Ruoff , G. H. Yu , Adv. Energy Mater. 2016, 6, 1600025.

[18] Y. Wen , K. He , Y. J. Zhu , F. D. Han , Y. H. Xu , I. Matsuda , Y. Ishii , J. Cumings , C. S. Wang , Nat. Commun. 2014, 5, 4033. 24893716 10.1038/ncomms5033

[19] X. J. Zhang , G. Zhu , M. Wang , J. B. Li , T. Lu , L. K. Pan , Carbon 2017, 116, 686–694.

[20] R. Raccichini , A. Varzi , S. Passerini , B. Scrosati , Nat. Mater. 2015, 14, 271–279. 25532074 10.1038/nmat4170

