# Supplementary material for: Covalent Triazine Frameworks via a Low‐Temperature Polycondensation Approach
Source: Angew Chem Int Ed Engl. 2017 Oct 9;56(45):14149–53. doi: 10.1002/anie.201708548 (PMC5698698; doi:10.1002/anie.201708548)
Supplement: Supplementary file 1 — Supplementary [file ANIE-56-14149-s001.pdf]

Angewandte Chemie
Eine Zeitschrift der Gesellschaft Deutscher Chemiker

Supporting Information

Covalent Triazine Frameworks via a Low-Temperature Polycondensation Approach

Kewei Wang, Li-Ming Yang, Xi Wang, Liping Guo, Guang Cheng, Chun Zhang, Shangbin Jin, Bien Tan,* and Andrew Cooper*

anie_201708548_sm_miscellaneous_information.pdf

1. Materials and Characterization

Benzene-1,4-dicarbonitrile, phosphorous oxychloride ($POCl_3$), thionyl chloride ($SOCl_2$), dimethyl sulfoxide (DMSO),cesium carbonate (Cs_2CO_3),potassium carbonate (K_2CO_3), tetrahydrofuran (THF), polyethylene glycol (PEG-400), methanol and absolute ethanol were analysis grade and purchased from National Medicines Corporation Ltd. of China. Triphenylamine, tris(4-iodophenyl)amine, 4-formyl-phenylboronic acid, 4,4'-biphenyl-dicarboxaldehyde, lithium bis(trimethylsilyl)azanide (1.0mol/L in THF), 1,4-phthalaldehyde, and bis(triphenylphosphine) palladium(II) dichloride were obtained from Aldrich Chemical Co. and used as received. Other reagents of analytical gradewere utilized without further purification.

Fourier-transformed infrared (FT-IR) spectra were collected on KBr disks in transmission mode using a Bruker Vertex 70 FTIR spectrometer. Ultraviolet–visible spectra were measured on a UV-VIS-NIR spectrophotometer (UV-3600, Shimadzu Japan). ^{13}C cross polarization magic angle spinning nuclear magnetic resonance (^{13}C CP/MAS NMR) spectra were recorded on a WB 400 MHz Bruker Avance II spectrometer with the contact time of 2 ms (ramp 100) and pulse delay of 3 s. Surface area, N_2 adsorption isotherms (77 K) and pore size distributions were measured using Micromeritics ASAP 2020 M surface area and porosity analyzer. Before analysis, the samples were degassed at 120 °C for 8 h under vacuum (10–5 bar). The surface areas were calculated based on nitrogen adsorption isotherms by Brunauer-Emmett-Teller (BET) or Langmuir analysis. Pore size distributions were calculated by DFT methods *via* the adsorption branch. The H_2 gas sorption isotherms were measured at 77.3 K and CO_2 gas isotherms were measured at 273 K. The pyrolysis was conducted in a SK2-4-13 tubular furnace (Yahua Furnace Co., Ltd, Wuhan). Elemental analysis (EA) measurements were performed on a VarioMicrocube Elemental Analyser (Elementar, Germany). Thermogravimetric analysis (TGA) was performed using a Perkin-Elmer Pyrisl TGA. The samples were heated at the rate of 10 °C/min under a nitrogen atmosphere up to 900 °C. The microstructural properties of electrode materials were characterized by X-ray diffraction using the Cu Kα radiation (λ = 1.5418 Å) (XRD, Philips X' Pert Pro). Transmission electron microscopy (TEM) images of the composites were obtained with Tecnai G2 F30 (FEI Holland) transmission electron microscope. Polymer morphologies were investigated with a FEI Sirion 200 field emission scanning electron microscope (FE-SEM). Before measurement, the samples were sputter coated with platinum. Atomic force microscopy (AFM) images were observed on a scanning probe microscopy

SPM-9700 instrument (Shimadzu Japan). The samples were dispersed in ethanol and ultrasonicated for 6 h at 25 °C and then two drops of suspension were taken onto the mica sheet and allowed to dry. Raman spectra were recorded on a confocal laser Raman spectrometer LabRAM HR800 (Horiba JobinYvon, France). X-ray photoelectron spectroscopy (XPS) analysis were conducted on an Axis Ultra DLD 600 W instrument (Shimadzu, Japan).

The photocatalytic experiments were performed under visible light irradiation (>420nm) with 300W Xe lamp (Perfectlight, PLS-SXE300). In this system, 50 mg photocatalysts were dispersed in 100ml aqueous solution which contained 10 ml TEOA. 3wt% Pt here was added as cocatalyst by in situ photodeposition method using H_2PtCl_6. The temperature of the solution was maintained at room temperature by the flow cooling water during the reaction. The hydrogen evolution was analyzed by gas chromatography (SHIMADZU, GC-2014 C) equipped with a thermal conductive detector (TCD) and 5A molecular sieve column using N_2 as the carrier gas.

The electrochemical properties of samples were measured on a Hokudo Denko charge/discharge instrument by using a 2032-type coin cell. Na metal foil was utilized as the reference and counter electrode. The electrolyte was 1M $NaPF_6$ in ethyl carbonate (EC) and diethyl carbonate (DEC) (EC: DEC=1:1 by volume). 15wt% acetylene black (AB) was used as a conductive agent and 5wt% polyvinylidene fluoride (PVDF) as a binder. A Cu foil was used as current collector. The cells were assembled in a glove box filled with pure argon gas. Galvanostatic discharge/charge measurements were performed in a potential range of 3 V-50 mV vs Na^+/Na.

Synthesis of model compound[S1]:

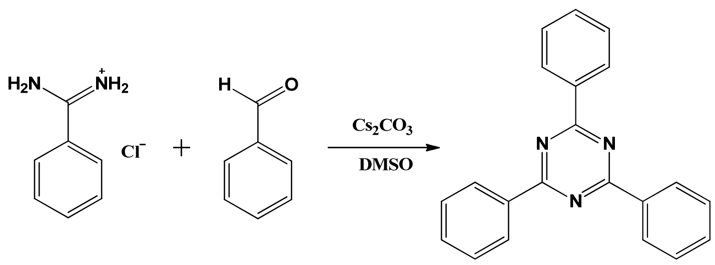

Model compound: 2,4,6-triphenyl-1,3,5-triazine was synthesized as reported.[S1]

^{1}H NMR (400MHz,$CDCl_3$): δ = 8.79 (d, 6H, J = 5.1, aromatic H), 7.57-7.64 (m, 9H, aromatic H), ^{13}C NMR (75 MHz, $CDCl_3$) δ 171.68, 136.27, 132.52, 128.99, 128.66.

Synthesis of terephthalamidine dihydrochloride[S2]:

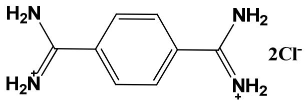

To a solution of benzene-1,4-dicarbonitrile (1.28 g, 10.0 mmol) in 20 mLTHF, 40 mL of 1 M $LiN(SiMe_3)_2$ solution was added dropwise in 30 min at 0 °C. The mixture was stirred at 25 °C for 3 h and then cooled to 0 °C. The reaction was quenched by careful addition of 6 M HCl–EtOH (40 mL) and the mixture was set aside overnight. The precipitate was then filtered, washed with Et_2O, and then the powder was recrystallized from H_2O–EtOH mixture. Yield: (2.26 g, 96%). ^{1}H NMR (400MHz, DMSO-d^6): δ = 9.63 (s, 4H, NH), 9.37 (s, 4H, NH), 8.03 (s, 4H, aromatic H), ^{13}C NMR (75 MHz, D_2O) δ =165.87, 132.89, 138.72.

Synthesis of tri(4-formylphenyl)amine[S3](Monomer 3: M3):

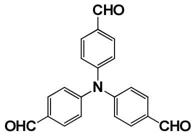

Phosphorous oxychloride (60.0 mL) was added dropwise to a stirred *N,N*-dimethylformamide (45.9 mL, 0.59 mol) at the temperature of ice water under nitrogen atmosphere. The mixture was stirred at 0 °C for another 1 h, and the solution turned pink. After the addition of triphenylamine (3.68 g, 15.0 mmol), the temperature was allowed to rise to 95 °C. After 72 h, the solution was poured into cold water. The resulting mixture was neutralized to pH = 7–8 with aqueous NaOH solution (20 wt %), and yellow solid was obtained. The solid was chromatographed on a silica gel column to produce 1.88 g of tri(4-formylphenyl)amine in 38% yield. ^{1}H NMR (400 MHz, $CDCl_3$): δ = 9.95 (s, 3H, −C*H*O), 7.85 (d, J = 6.3 Hz, 6H, Ar−*H*), 7.26 (d, J = 6.3 Hz, 6H, Ar−*H*); ^{13}C NMR (75 MHz, $CDCl_3$) δ =190.47, 151.20, 132.61, 131.51, 124.60.

Synthesis of tri(4-formylbiphenyl)amine[S3] (Monomer 4: M4):

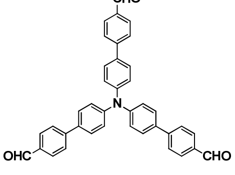

Tris(4-iodophenyl)amine (623 mg, 1.00 mmol) and 4-formylphenylboronic acid (540 mg, 3.60 mmol)

were dissolved in 20 mL of tetrahydrofuran. Aqueous solution of potassium carbonate (10.0 mL, 2.0 mol L^{-1}) was added into the solution under nitrogen atmosphere. After the addition of bis(triphenylphosphine)palladium(II) dichloride (105 mg), the mixture was refluxed for 12 h. The solution was extracted twice with dichloromethane (3 × 100 mL). The obtained organic layer was washed with plenty of water and the solvent was removed at reduced pressure. The residue was chromatographed on a silica gel column to give yellow solid 0.47 g tri(4-formylbiphenyl)amine with 85 % yield. ^{1}H NMR (400 MHz, $CDCl_3$): δ =10.05 (s, 3H, −C*H*O), 7.96 (d, *J* = 6.3 Hz, 6H, Ar−*H*), 7.76 (d, *J* = 6.0 Hz, 6H, Ar−*H*), 7.61 (d, *J* = 6.3 Hz, 6H, Ar−*H*), 7.29 (d, *J* = 6.3 Hz, 6H, Ar−*H*). ^{13}C NMR (75 MHz, $CDCl_3$) δ = 191.80, 147.47, 146.31, 134.98, 134.46, 130.39, 128.40, 127.11, 124.66.

Synthesis of CTF-HUST-1. 1,4-Phthalaldehyde (67.2 mg, 0.5 mmol), terephthalamidine dihydrochloride (235.2 mg, 1.0 mmol), and cesium carbonate (716.8 mg, 2.2 mmol) were added to a solution of DMSO (5.0 mL) and H_2O (0.2 mL) in 25 mL round-bottom flask. The mixture was heated at 60 °C for 12 h, then heated at 80 °C, and then 100°C for 12 h, separately, before being heated at 120 °C for 3 days to yield a yellow solid. The resulting precipitate was washed with dilute HCl (3 × 10 mL) to remove the salt and residual cesium carbonate, and washed with water (3 × 10 mL), acetone (3 × 10 mL), and THF (3 × 10 mL), before drying at 80 °C under vacuum for 12 h to yield CTF-HUST-1 as a yellow power (209.9 mg, 91% yield).

Amplified synthesis of CTF-HUST-1. 1,4-Phthalaldehyde (2.01 g,15.0 mmol), terephthalamidine dihydrochloride (7.05 g, 30.0 mmol), cesium carbonate (19.55 g, 60.0 mmol) were added to a solution of DMSO (100.0 mL) and H_2O (4.0 mL) in 500 mL round-bottomed. The mixture were strirred by mechanical agitation at 60 °C for 12 h, then heated at 80 °C, 100°C for 12 h separately, and heated at 120 °C for 3 days, yielding a canary yellow solid. The resulting precipitate was washed with diluted HCl (3×100 mL) to remove the salt and residual cesium carbonate, and washed with water (3×300 mL), acetone (3×300 mL) and THF (3×300 mL), dried at 80 °C under vacuum for 12 h to yield CTF-HUST-1 as a yellowish power (5.61 g, 81% yield).

Synthesis of CTF-HUST-2. Similar to CTF-HUST-1,4,4'-Biphenyldicarboxaldehyde (105.1 mg, 0.5 mmol), terephthalamidine dihydrochloride (235.2 mg, 1.0 mmol), cesium carbonate (716.8 mg, 2.2 mmol)

were added to a solution of DMSO (5.0 mL) and H_2O (0.2 mL) in 25 mL round-bottomed. The mixture was heated at 60 °C for 12 h, then heated at 80 °C, 100°C for 12 h separately, and heated at 120 °C for 3 days, yielding a yellowish solid. The resulting precipitate was washed with diluted HCl (3×10 mL) to remove the salt and residual cesium carbonate, and washed with water (3×10 mL), acetone (3×10 mL) and THF (3×10 mL), dried at 80 °C under vacuum for 12 h to yield CTF-HUST-2 as a power (249.9 mg, 93% yield).

Synthesis of CTF-HUST-3. Tri(4-formylphenyl)amine(82.3 mg, 0.25 mmol), terephthalamidine dihydrochloride (176.4 mg, 0.75 mmol), cesium carbonate (537.6 mg, 1.65 mmol) were added to a solution of DMSO (5.0 mL) and H_2O (0.2 mL) in 25 mL round-bottomed. The mixture was heated at 60 °C for 12 h, then heated at 80 °C, 100°C for 12 h separately, and heated at 120 °C for 3 days, yielding a yellow solid. The resulting precipitate was washed with diluted HCl (3×10 mL) to remove the salt and residual cesium carbonate, and washed with water (3×10 mL), acetone (3×10 mL) and THF (3×10 mL), dried at 80 °C under vacuum for 12 h to yield CTF-HUST-3 as a power (182.5 mg, 89% yield).

Synthesis of CTF-HUST-4. Similar to CTF-HUST-3, tri(4-formylbiphenyl)amine (139.4 mg, 0.25 mmol), terephthalamidine dihydrochloride (176.4 mg, 0.75 mmol), cesium carbonate (537.6 mg, 1.65 mmol) were added to a solution of DMSO (5.0 mL) and H_2O (0.2 mL) in 25 mL round-bottomed. The mixture was heated at 60 °C for 12 h, then heated at 80 °C, 100° C for 12 h separately, and heated at 120 °C for 3 days, yielding a yellow solid. The resulting precipitate was washed with diluted HCl (3×10 mL) to remove the salt and residual cesium carbonate, and washed with water (3×10 mL), acetone (3×10 mL) and THF (3×10 mL), dried at 80 °C under vacuum for 12 h to yield CTF-HUST-4 as a power (235.9 mg, 90% yield).

Pyrolysis of CTF-HUSTs. The CTF-HUSTs (0.5 g) was heated to 800 °C at a heating rate of 5 °C min^{-1} and carbonized at 800 °C for 3 h under inert atmosphere (nitrogen flow) in a tubular furnace, then cooled to room temperature naturally to obtain *p*-CTF-HUSTs. The carbonization yields of *p*-CTF-HUST-1, *p*-CTF-HUST-2, *p*-CTF-HUST-3 and *p*-CTF-HUST-4 are 36.7%, 42.8%, 39.39% and 35.5% respectively.

2. Supporting Figures

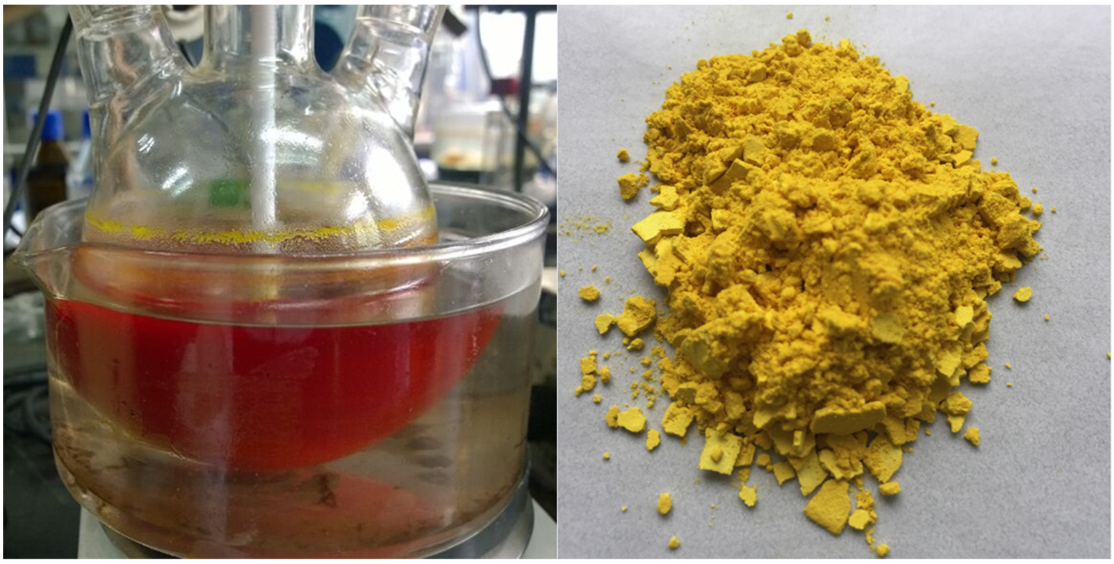

Figure S1. Amplification experiment of CTF-HUST-1

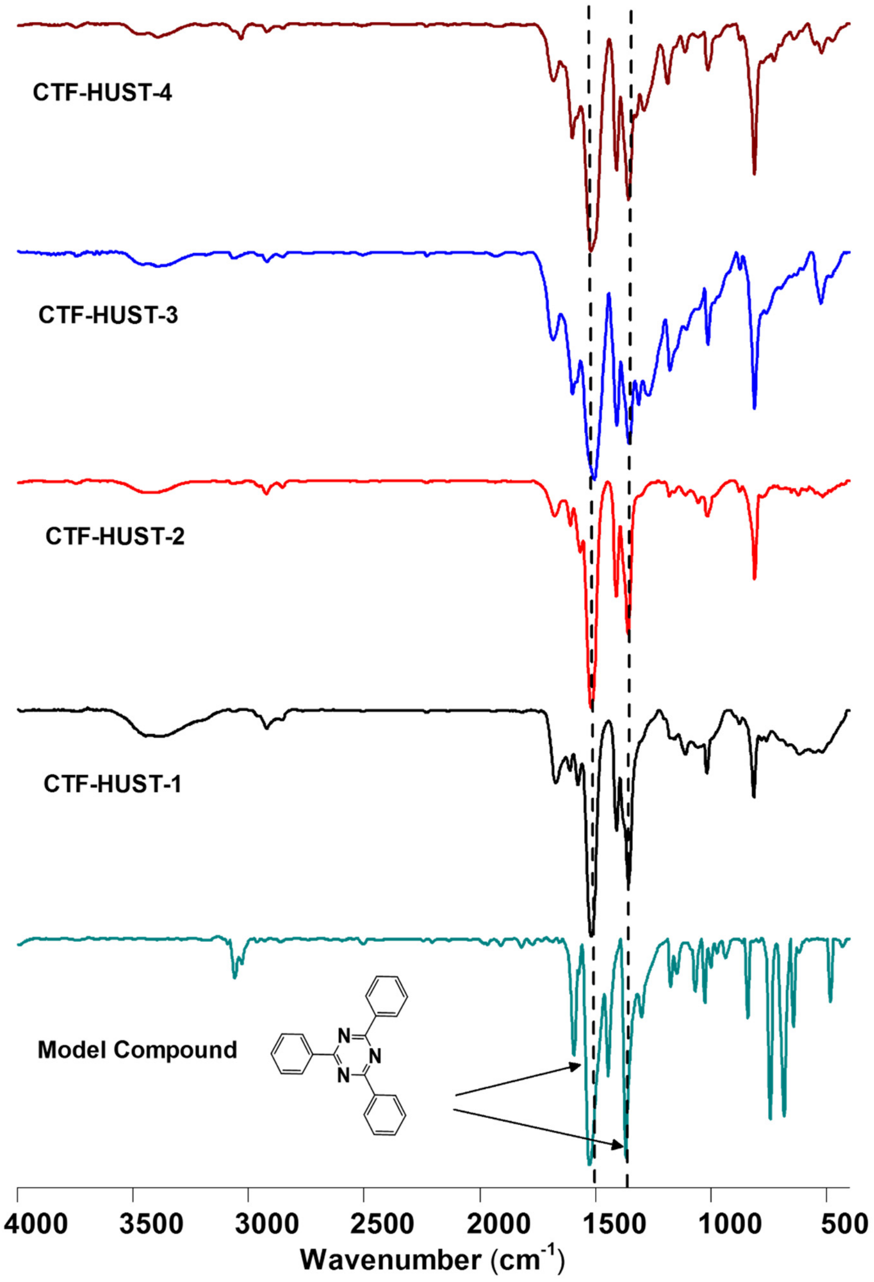

Figure S2. FT-IR of CTF-HUSTs and model compound.

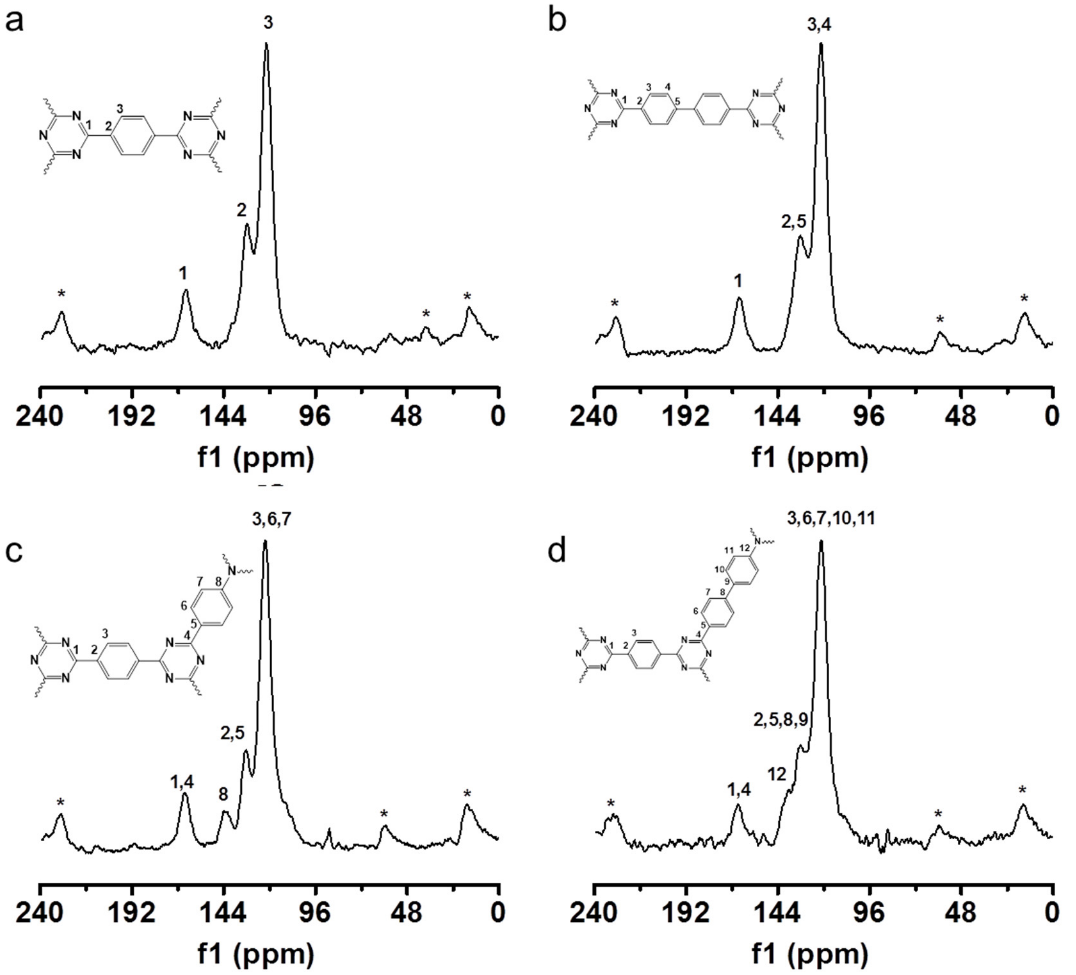

Figure S3. ^{13}C CP-MAS solid state NMR spectra of CTF-HUST-1 (a), CTF-HUST-2 (b), CTF-HUST-3 (c), CTF-HUST-4 (d)

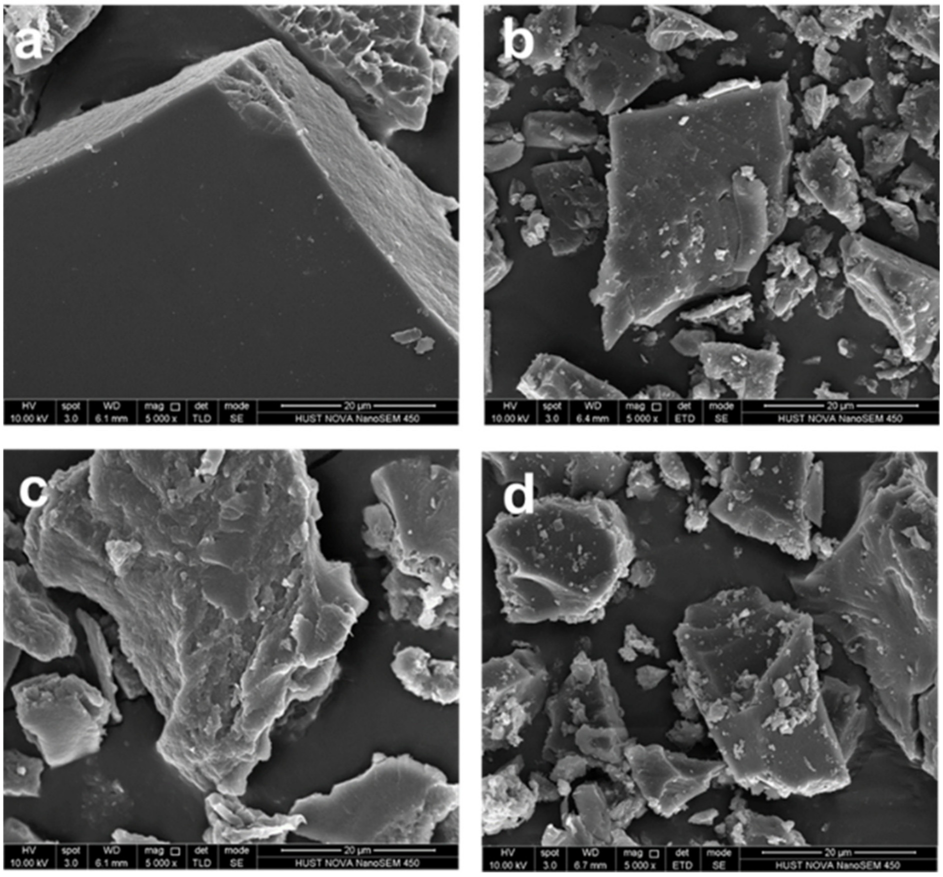

Figure S4. SEM image of CTF-HUST-1 (a), CTF-HUST-2 (b), CTF-HUST-3 (c), and CTF-HUST-4 (d)

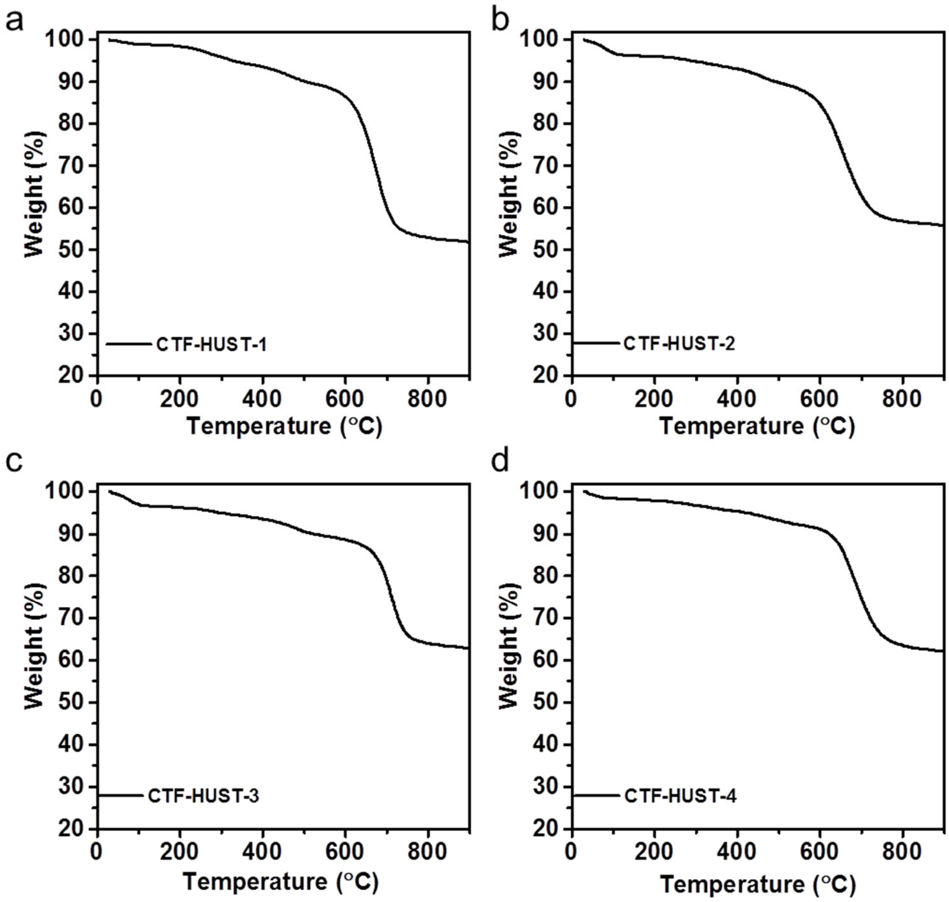

Figure S5.TGA curves of a) CTF-HUST-1, b) CTF-HUST-2, c) CTF-HUST-3, d) CTF-HUST-4

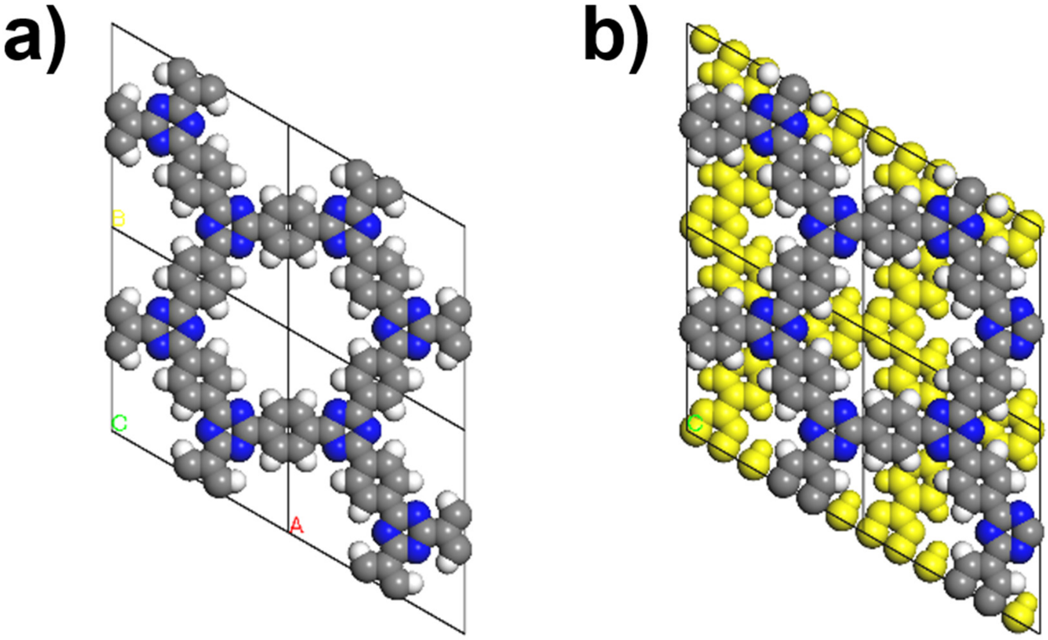

Figure S6 Unit cell of AA stacking and CTF-HUST-1 and AB stacking of CTF-HUST-1

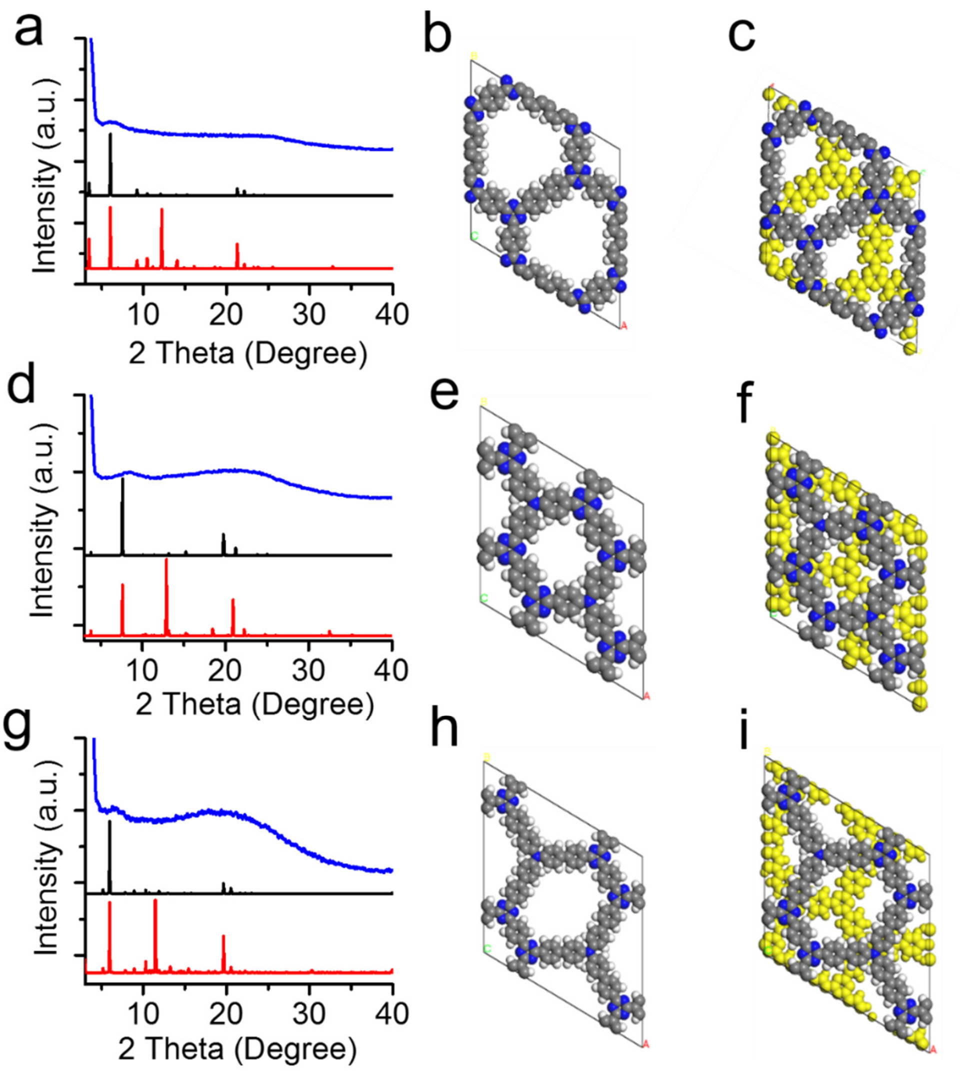

Figure S7. PXRD pattern of experimental (blue), simulated AA stacking (black) and simulated AB stacking (red) of CTF-HUST-2 (**a**), CTF-HUST-3 (**d**) and CTF-HSUT-4 (**g**); Unit cell of AA stacking and CTF-HUST-2 (**b**), CTF-HUST-3 (**e**), and CTF-HSUT-4 (**h**); AB stacking of CTF-HUST-2 (**c**), CTF-HUST-3 (**f**), and CTF-HSUT-4 (**i**).

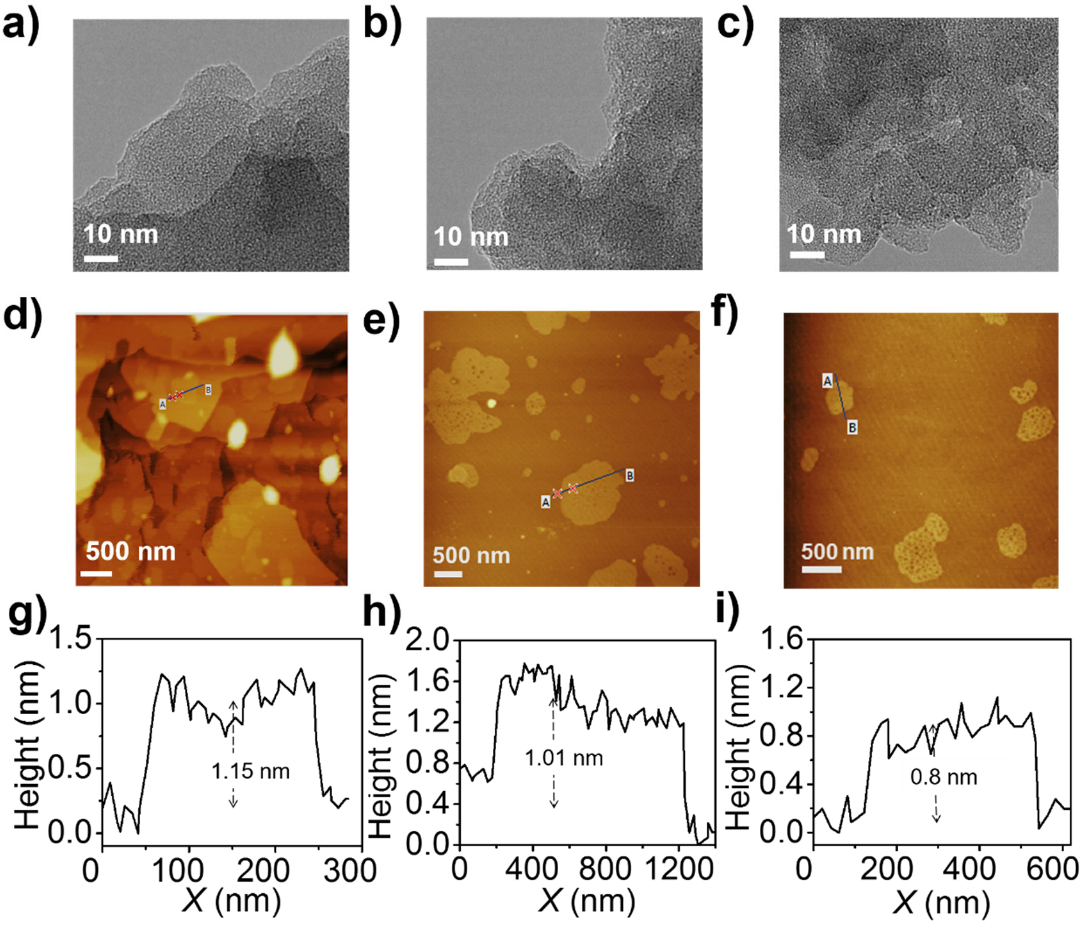

Figure S8. (a-c) High-Resolution TEM images of (a) CTF-HUST-2, (b) CTF-HSUT-3 and (c) CTF-HSUT-4; (d-e) Atomic force microscopy topography of (d) CTF-HUST-2, (e) CTF-HUST-3 and (f) CTF-HUST-4 on a mica sheet; (g-i) Height profile of AFM of the corresponding edge height of (g) CTF-HUST-2, (h) CTF-HUST-3 and (i) CTF-HUST-4.

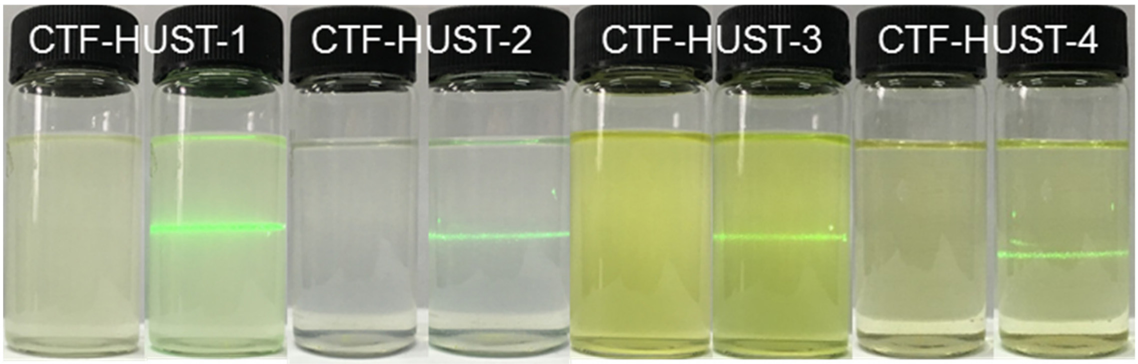

Figure S9. Photographs of CTF-HUSTs suspension and Tyndall effect observed with a concentration of 0.1 mg mL^{-1} in ethanol.

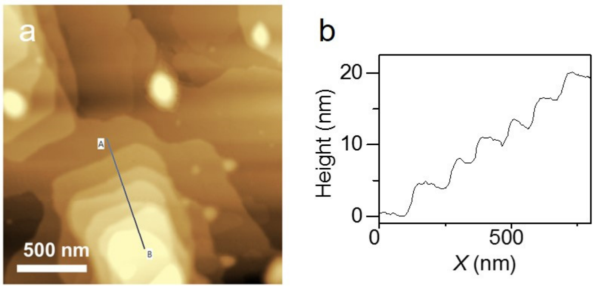

Figure S10. Atomic force microscopy images of CTF-HUST-1 with multilayers stacking with each other.

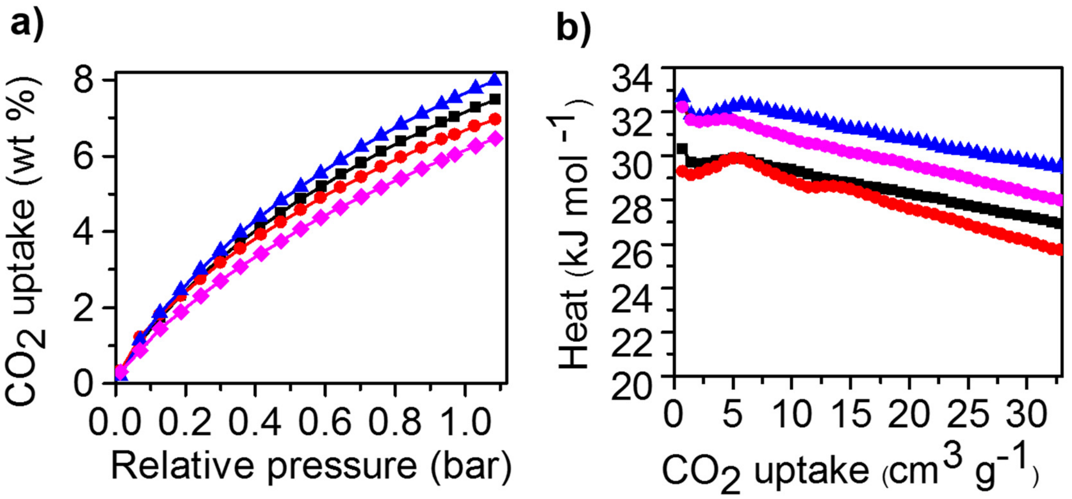

Figure S11 (a) CO_2 sorption curves of CTF-HUST-1(black curve), CTF-HUST-2 (red curve), CTF-HUST-3 (blue curve) and CTF-HUST-4 (magenta curve) at 298 K; (b) adsorption heat of CO_2

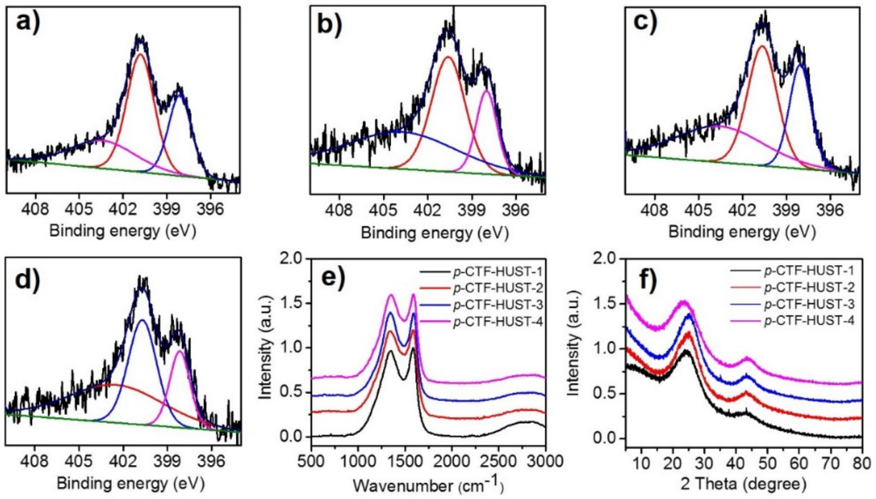

Figure S12. (a-d) X-ray photoelectronic spectroscopy of (a) *p*-CTF-HUST-1, (b) *p*-CTF-HUST-2, (c) *p*-CTF-HUST-3 and(d) *p*-CTF-HUST-4; (e) Raman spectra of *p*-CTF-HUSTs; (f) Powder X-ray diffraction of *p*-CTF-HUSTs. The XPS spectra show three types of nitrogen peaks in p-CTF-HUSTs with binding energies at 398.1 eV, 400.5 eV, and 403.7 eV, which were assigned to pyridinic nitrogen, pyrrolic nitrogen, and pyridine N-oxide nitrogen (Figure S12a-d) respectively.[S4] The Raman spectra showed that there are mainly two bands at 1354 cm^{-1} and 1584 cm^{-1}, which are attributed to D and G bands, respectively (Figure S12e).[S5] The values of I_D/I_G are 0.97, 0.98, 1.0, and 0.99, for *p*-CTF-HUST-1, *p*-CTF-HUST-2, *p*-CTF-HUST-3 and *p*-CTF-HUST-4, respectively. A broad band centered at 2800 cm^{-1} was also observed indicating the existence of layered graphene-like materials. The formation of the graphene-like layered structures was further evidenced by the experimental PXRD patterns of the carbonized samples (Figure S12f). Two broad peaks were clearly observed, which is consistent with carbonaceous materials with graphene-like structures.[S6] The peaks at 22°–25° derived from the diffraction of the layer structures, whereas 43° originated from the disordered carbon structures.

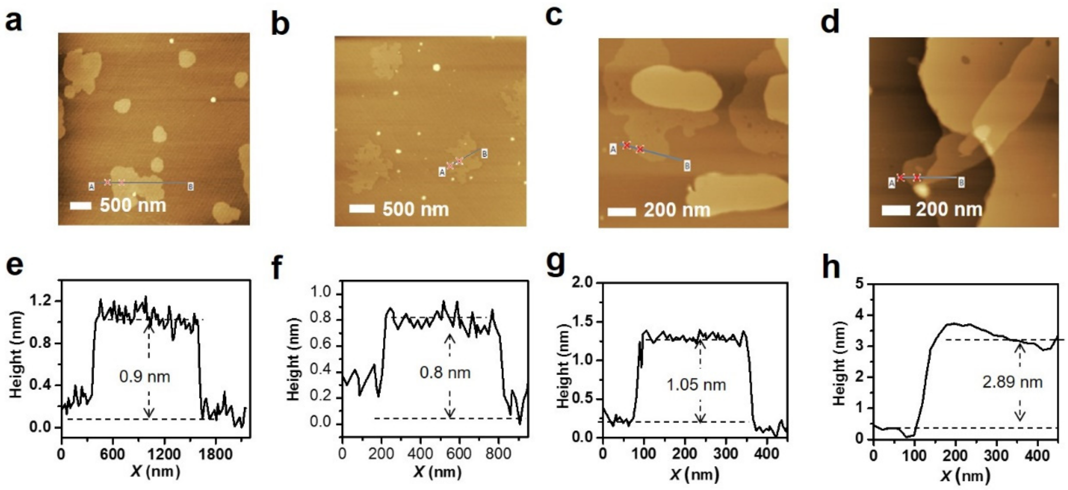

Figure S13. (**a-d**) Atomic force microscopy images: (**a**) *p*-CTF-HUST-1, (**b**) *p*-CTF-HUST-2, (**c**) *p*-CTF-HUST-3 and (**d**) *p*-CTF-HUST-4; (e-h) Height profile of AFM of the corresponding edge height of (**e**) *p*-CTF-HUST-1, (**f**) *p*-CTF-HUST-2, (**g**) *p*-CTF-HUST-3 and (**h**) *p*-CTF-HUST-4.

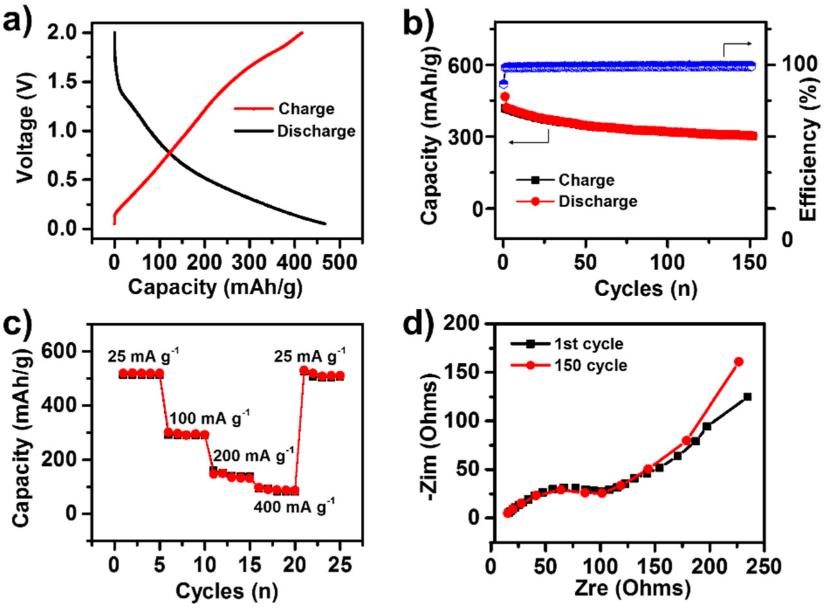

Figure S14 (a) Galvanostatic charge/discharge profiles at 50 mA g^{-1} in the first cycle; (b) cyclic performance at a current density of 50 mA g^{-1}; (c) Rate performance at different current densities ranging from 25 to 400 mA g^{-1}; (d) Nyquist plots of the initial state and after 150 cycles. *p*-CTF-HUST-4 holds a high initial Coulombic efficiency of approximately 89.1% (Figure S14b). We also found *p*-CTF-HUST-4 to be a stable SIB anode material. Even after 150 cycles, it delivered 303 mAh g-1 with a slight decrease in capacity and a high Coulombic efficiency of 99.9%. *p*-CTF-HUST-4 also exhibited excellent rate capabilities, with high capacity of 514 mAh g^{-1} at 25 mA g-1, 291 mAh g^{-1} at 100 mA g^{-1}, and 97 mAh g^{-1} at 400 mA g^{-1}, as shown in Figure S14c. These results indicate that there are some remarkable features of *p*-CTF-HUST-4 as an anode material for Na^+ storage, such as good compatibility with electrolyte, and significant improvement of the sodium transportation kinetics and the electron conductivity, thus enhancing the reversible capacity and rate capability. The electrode kinetics investigated by electrochemical impedance spectrum (EIS) are shown in Figure S14d. In high frequency areas, it is found that the diameter of the semicircle at the initial state and after 150 cycle is nearly the same, suggesting the low con-tact/charge-transfer impedances. It should be noted that there is a steep low-frequency tail after 150 discharging/charging, indicating high sodium ions diffusivity.

NMR spectra

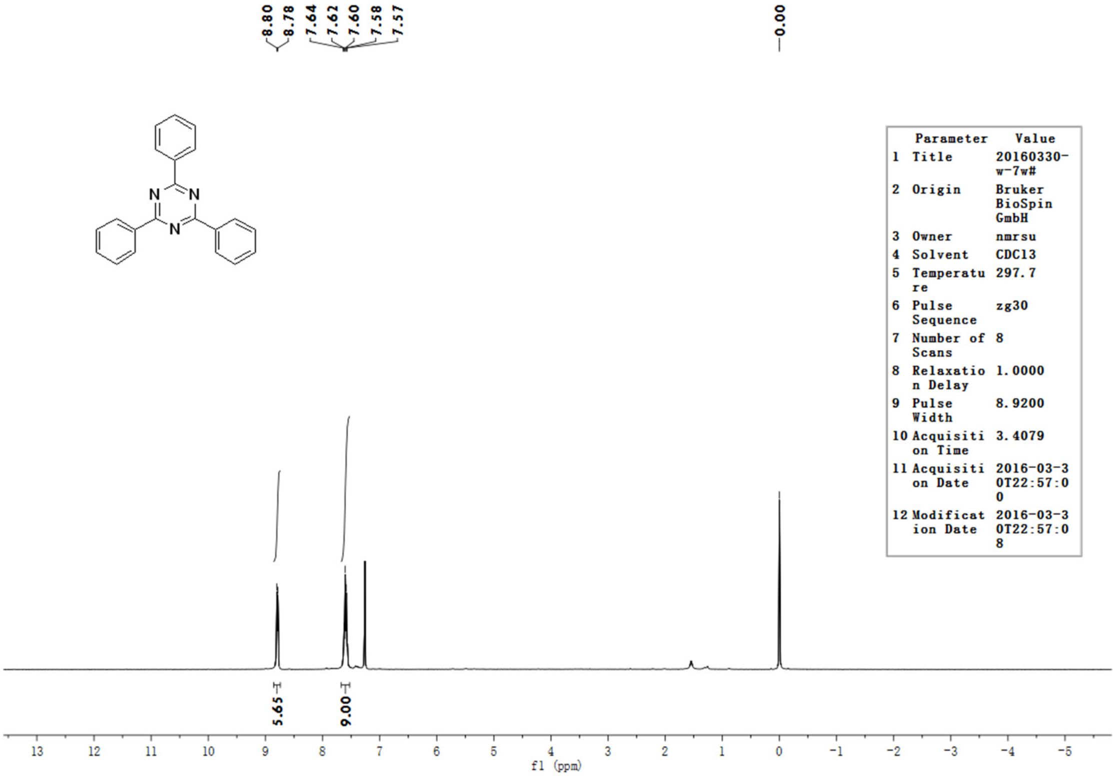

Figure S15. ^{1}H NMR spectrum of model compound

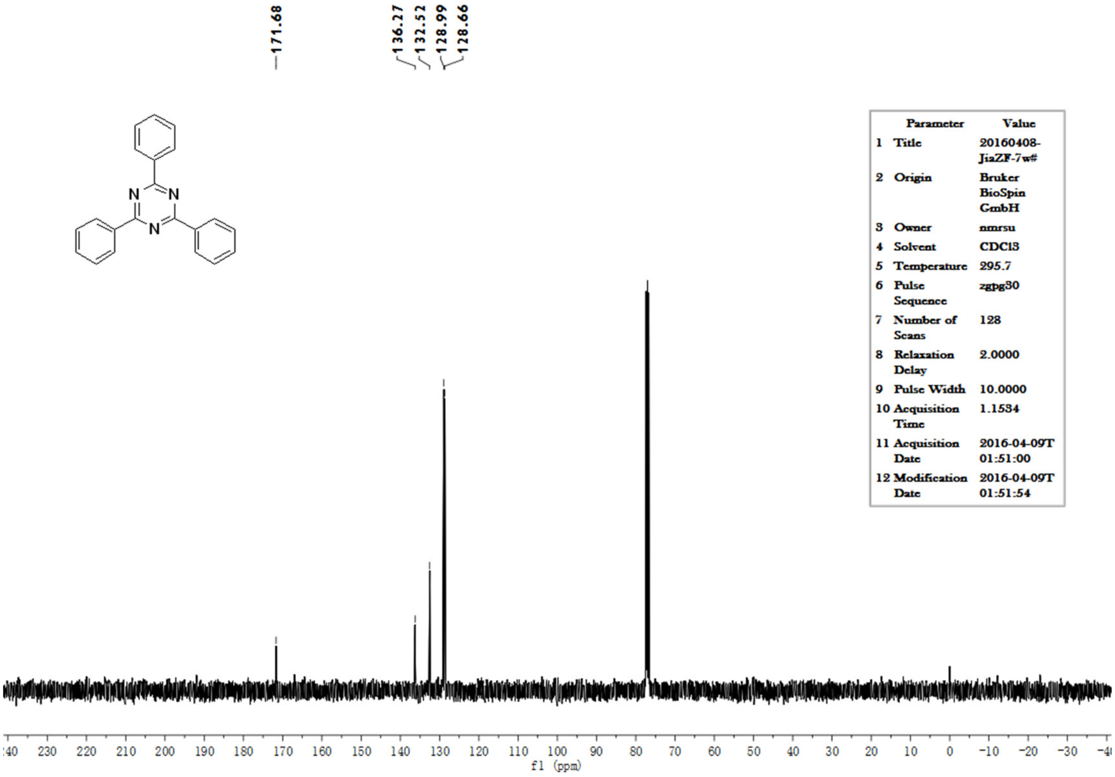

Figure S16. ^{13}C NMR spectrum of model compound

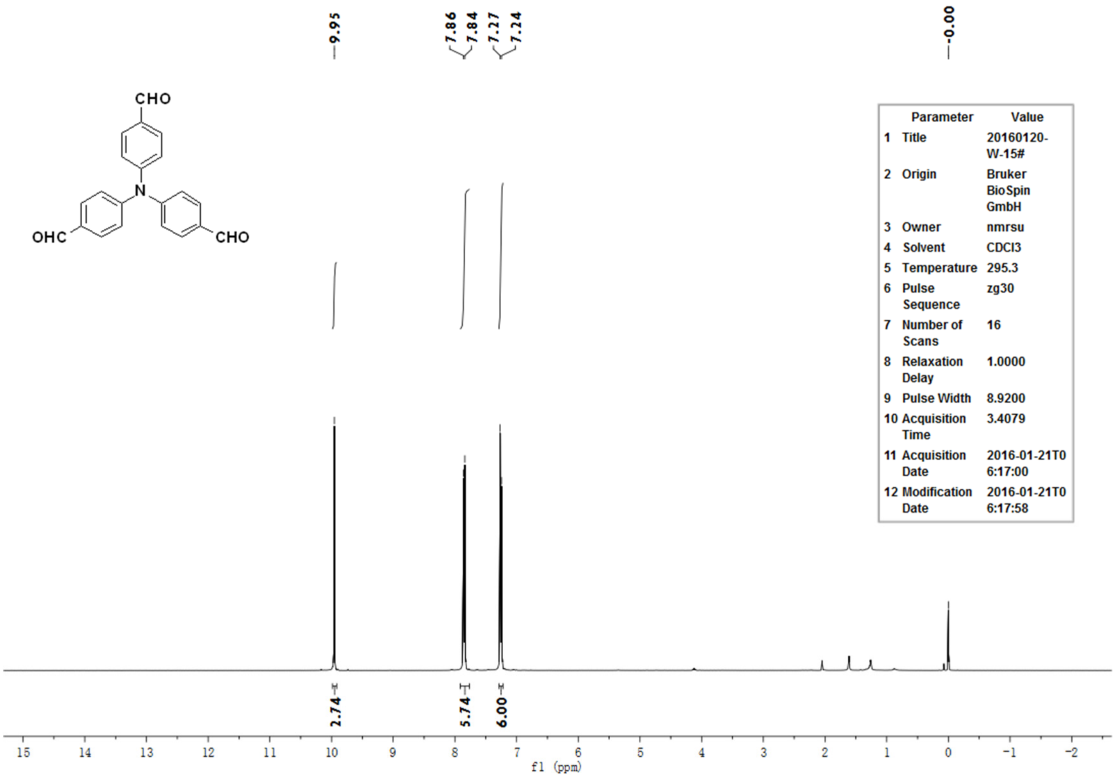

Figure S17. ^{1}H NMR spectrum of **M3**

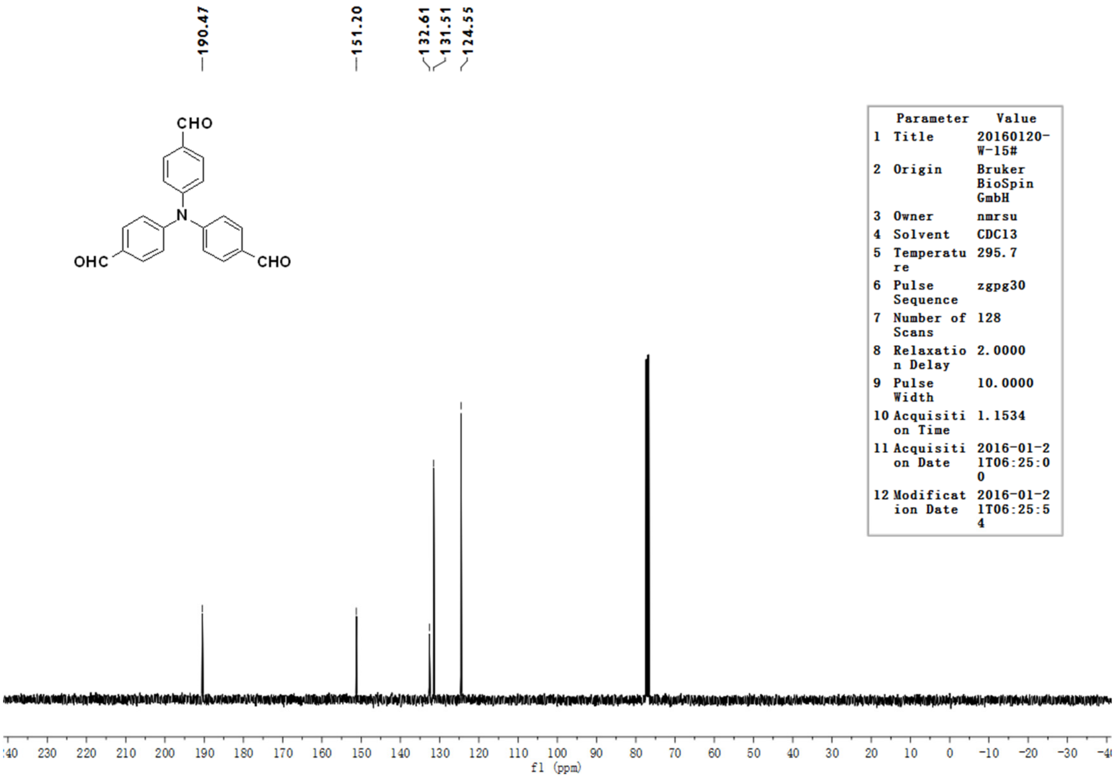

Figure S18. ^{13}C NMR spectrum of **M3**

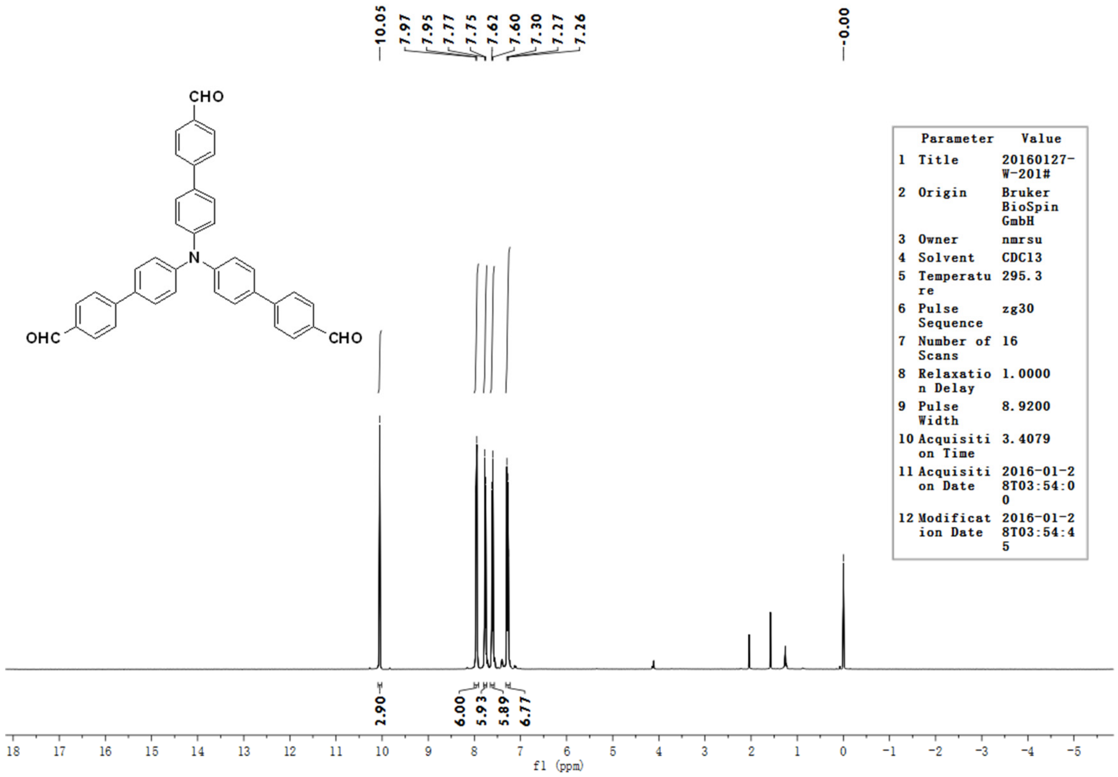

Figure S19.^{1}H NMR spectrum of **M4**

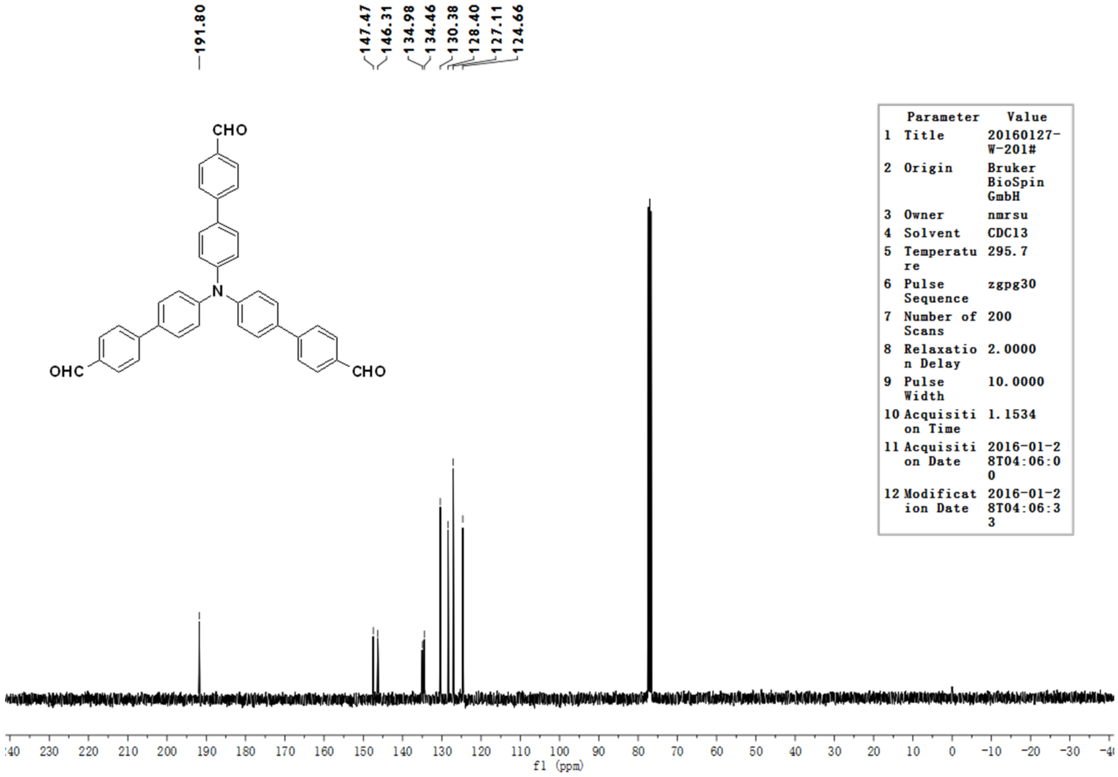

Figure S20.^{13}C NMR spectrum of **M4**

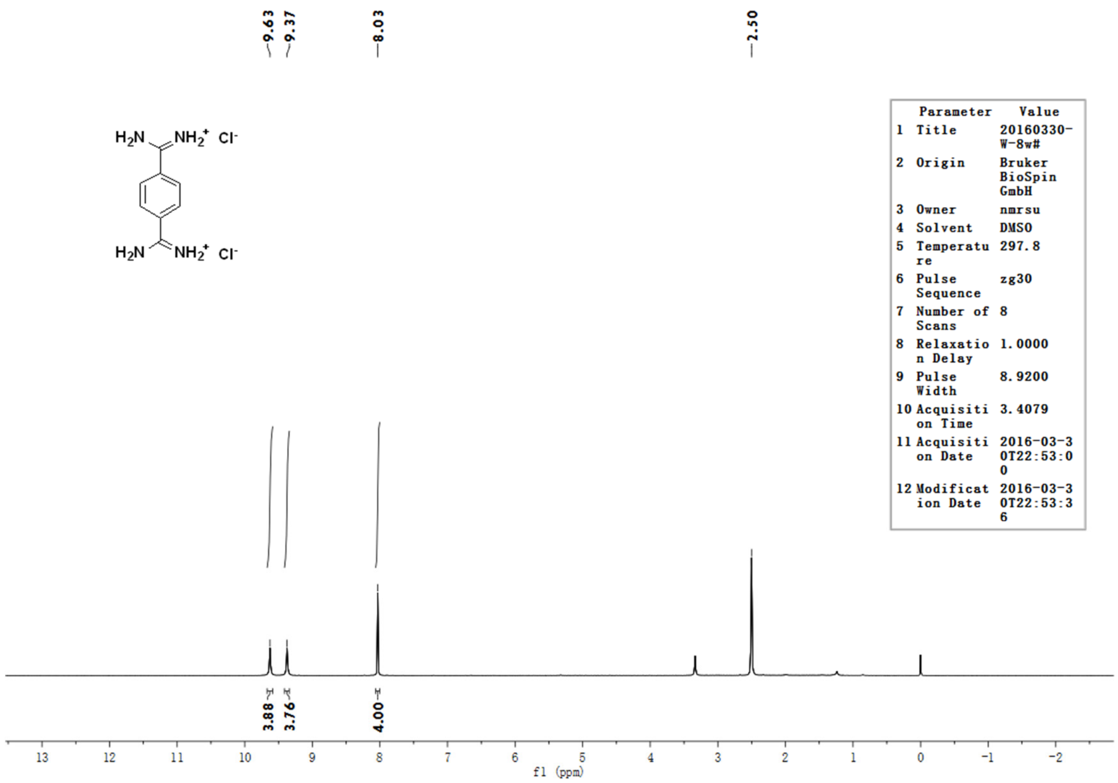

Figure S21.^{1}H NMR spectrum of terephthalamidine dihydrochloride

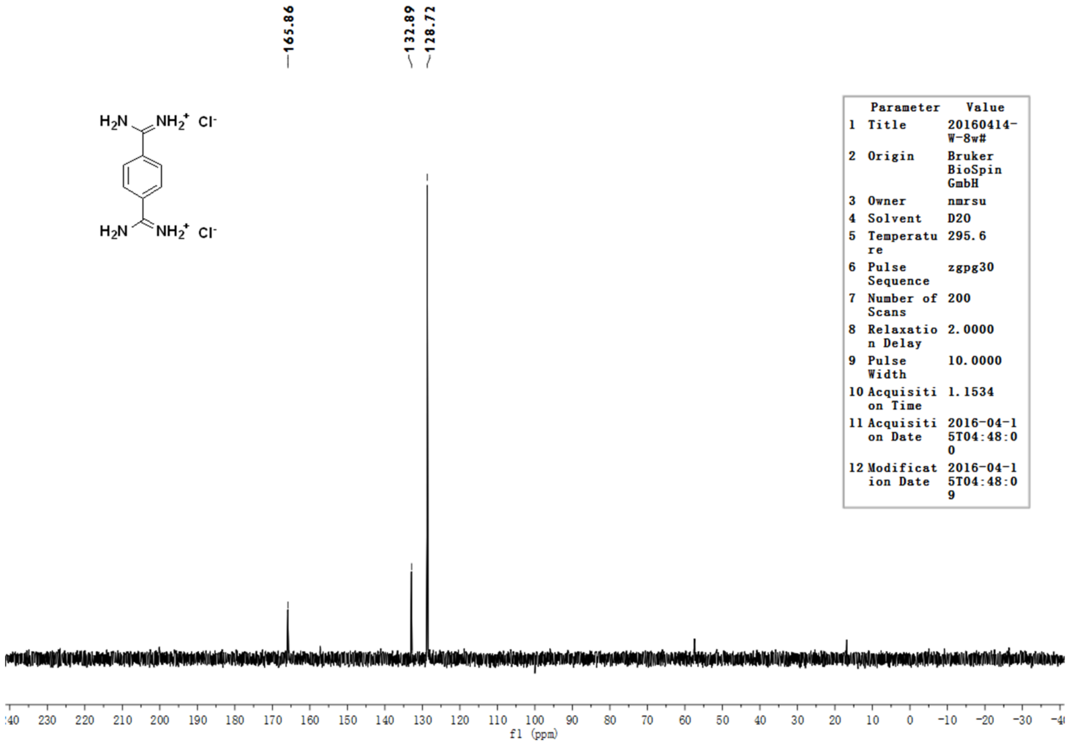

Figure S22.^{13}C NMR spectrum of terephthalamidine dihydrochloride

3. Supporting Tables

Table S1. Synthesis of the CTF-HUST-1 under variable conditions

Solvent	Temperature (°C)	Reaction time (day)	S_{BET} ($m^2 g^{-1}$)
DMF (3mL)	120	3	30
DMF+mesitylene (1.5mL +1.5mL)	120	3	38
DMF+o-dichlorobenzene (1.5mL +1.5mL)	120	3	12
DMF+Dioxane (1.5mL +1.5mL)	120	3	28
Dioxane (3mL)	reflux	3	53
Dioxane+mesitylene (1.5mL +1.5mL)	120	3	0
DMAC (3mL)	120	3	12
DMAC+mesitylene (1.5mL +1.5mL)	120	3	29
DMAC+o-dichlorobenzene (1.5mL +1.5mL)	120	3	34
DMAC+ Dioxane (1.5mL +1.5mL)	120	3	278
NMP (3mL)	120	3	64
NMP+mesitylene (1.5mL +1.5mL)	120	3	17
NMP+o-dichlorobenzene (1.5mL +1.5mL)	120	3	42
NMP+Dioxane (1.5mL +1.5mL)	120	3	50
o-dichlorobenzene (3mL)	120	3	0
DMSO (3mL)	120	3	663
DMSO+mesitylene (1.5mL +1.5mL)	120	3	346
DMSO+ Dioxane (1.5mL +1.5mL)	120	3	148
DMSO+o-dichlorobenzene (1.5mL +1.5mL)	120	3	151
DMSO+H_2O (1.5mL +1.5mL)	120	3	0
H_2O (3mL)	reflux	3	No product

Table S2. Elemental analysis of CTF-HUSTs

EA (wt%)	CTF-HUST-1		CTF-HUST-2		CTF-HUST-3		CTF-HUST-4	
	Exp.	Cal.	Exp.	Cal.	Exp.	Cal.	Exp.	Cal.
C	67.7	73.8	69.2	77.2	68.7	78.0	73.8	82.8
H	4.6	4.7	4.3	4.8	4.5	4.8	4.6	4.9
N	18.6	21.5	15.4	18.0	16.7	17.2	12.8	12.3

Table S3. The optimized simulation lattice parameters.

	**CTF-HUST-1**	**CTF-HUST-2**	**CTF-HUST-3**	**CTF-HUST-4**
Space group	P6/mmm (#191)	P6/mmm (#191)	P6/mmm (#191)	P6/mmm (#191)
Lattice parameter	a=b=14.48041<br>c=3.62174<br>α=β=90<br>γ=120	a=b=29.26670<br>c=4.17055<br>α=β=90<br>γ=120	a=b=26.89862<br>c=4.49267<br>α=β=90<br>γ=120	a=b=34.33545<br>c=4.51542<br>α=β=90<br>γ=120
Total number of atoms per primitive cell	42	156	158	218
K-POINTS	1×1×2	1×1×1	1×1×1	1×1×1

Table S4. Summary of surface area, porosity and gas adsorption of CTF-HUSTs.

Samples	S_{BET}[a] $m^2\ g^{-1}$	S_L[b] $m^2\ g^{-1}$	M.A.[c] $m^2\ g^{-1}$	PV[d] $cm^3\ g^{-1}$	H_2 uptake $cm^3\ g^{-1}$	CO_2 uptake $cm^3\ g^{-1}$	CO_2/N_2
CTF-HUST-1	663	804	464	0.32	0.82	12.65	42.5
CTF-HUST-2	757	931	482	1.18	1.28	12.41	28.4
CTF-HUST-3	807	1003	490	0.45	1.17	13.91	37.7
CTF-HUST-4	764	972	415	0.78	1.04	11.67	34.8

a) Surface area calculated from nitrogen adsorption isotherms at 77.3 K using BET equation; b) Surface area calculated from nitrogen adsorption isotherms at 77.3 K using Langmuir equation; c) t-Plot micropore area; d) Pore volume calculated from nitrogen isotherm at P/P0 = 0.995, 77.3 K.

4. The simulation and optimized configuration of CTF-HUSTs:

The starting configurations were built by Accelrys' Materials Studio modelling software. The theoretical crystalline structures were simulated by using Reflex module of the Materials Studio program. The electronic structure and total energy were calculated using density functional theory (DFT)[S7] via the plane-wave pseudopotential (PWPP) technique implemented in the Vienna *ab initio* simulation package (VASP).[S8] The projector-augmented wave (PAW)[S9,S10] method was used to represent the ion-electron interaction. The generalized gradient approximation (GGA)[S11] expressed by the PBE functional[S12] and a 400 eV cutoff for the plane-wave basis set were adopted in all calculations. The convergence threshold was set as 10^{-6} eV in energy and the atoms were relaxed toward equilibrium until the Hellmann−Feynman forces were less than 10^{-2} eV/Å. Brillouin-zone integration was performed with a Gaussian broadening of 0.1 eV during all relaxations. Due to the stacking of layers in CTFs, it is important to account for dispersion effects, thus, DFT+D3 was used to afford the dispersion interactions between adjacent layers of CTFs.[S13] Due to the large number of atoms in each CTF system, we use the Γ-point in all VASP calculations for the balance between accuracy and computational cost. Due to the large number of atoms in each CTF system, these calculations can be conducted with a single *K*-point at the Γ-point. Given that the size of system and the balance between accuracy and computational cost, the *K*-point sampling was selected (Table S3) to build models of possible perfectly ordered network structures to compare with our experimental data. The unit cell structures were built with hexagonal geometry and the theoretical XRD was simulated by Materials Studio software (Figure 1a and Figure S6-S7). The unit cells were constructed using hexagonal geometry with P6/mmm space groups. As shown in Table S3, the optimized lattice parameters for CTF-HUST-1 are $a = b = 14.4804$ Å, $c = 3.6217$ Å, whereas the parameters of CTF-HUST-2, CTF-HUST-3 and CTF-HUST-4 are $a = b = 29.2667$ Å, $c = 4.1705$ Å; $a = b = 26.8986$ Å, $c = 4.4926$ Å and $a = b = 24.3354$ Å, $c = 4.5154$ Å, respectively. The simulated AA stacking and AB stacking structures are shown in Figure 1 and Figure S6-S7. The absence of any features in the 2 Theta = 10–15° range suggests that AA layer stacking are perhaps more likely, but the experimental features are too broad to allow any definitive model. For CTF-HUST-1, the proposed structure is most consistent with the previously reported CTF-1, which was reported to have AA stacking (Figure 2a, black curve).[S14] Indeed, the PXRD data for CTF-HUST-1 is quite similar to that reported for CTF-1,[S14] although for CTF-HUST-1 the peaks are broader. CTF-HUST-2, CTF-HUST-3 and CTF-HUST-4 form a new series of

structures with geometries that are dissimilar from any previously reported CTFs owing to their distinctive linkage mode (Figure S7). Unlike previous CTFs prepared by self-cyclization of nitrile building blocks, our new method involves two types of building blocks—an aldehyde and an amidine—which undergo a multi-step condensation process during the polymerization, as shown in Scheme 1a. Two equivalents of terephthalamidine monomers to aldehyde monomers are required in the cyclization reaction, allowing for CTFs with mixed functionalities. Hence, CTF-HUST-1 (and the earlier CTF-1) consists of one type of pore,[S14] whereas there are theoretically two types of pores in CTF-HUST-2, CTF-HUST-3 and CTF-HUST-4 (Scheme 1). CTF-HUST-1 has a theoretical pore size at 12 Å according to the perfectly crystalline model. By contrast, CTF-HUST-2 consists of two theoretical pore sizes at 12.0 Å and 15.0 Å. Likewise, CTF-HUST-3 consists of two theoretical pore size at 12.0 Å and 10.0 Å, and CTF-HUST-4 has two theoretical pore sizes of 12.0 Å and 14.7 Å, respectively (Scheme 1).

CTF-HUST-1

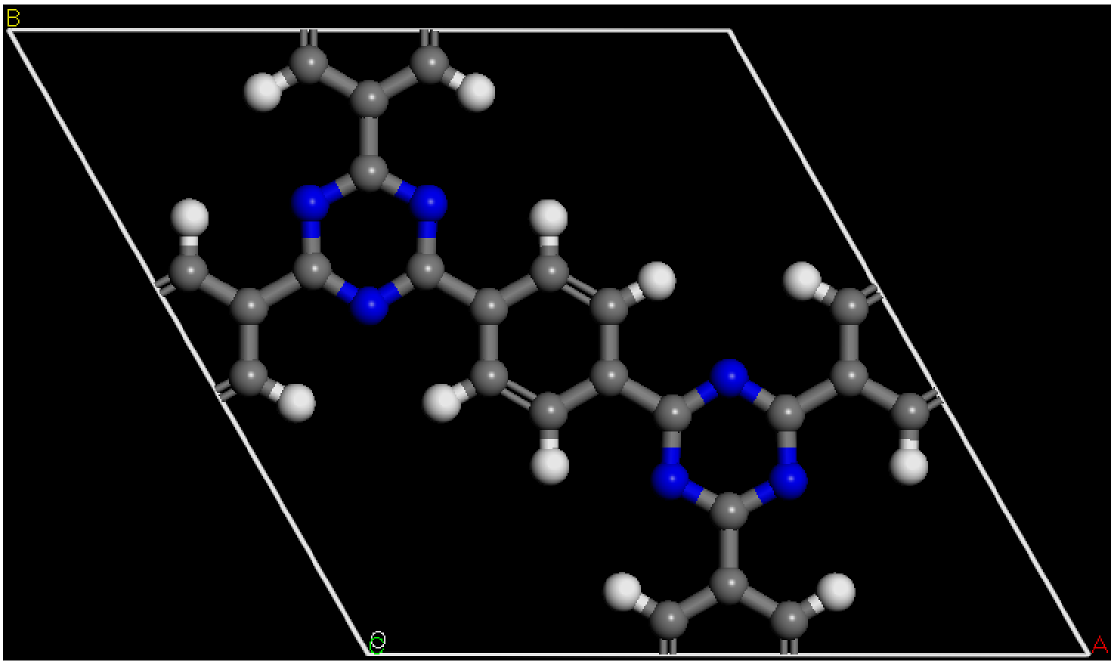

CTF-HUST-2

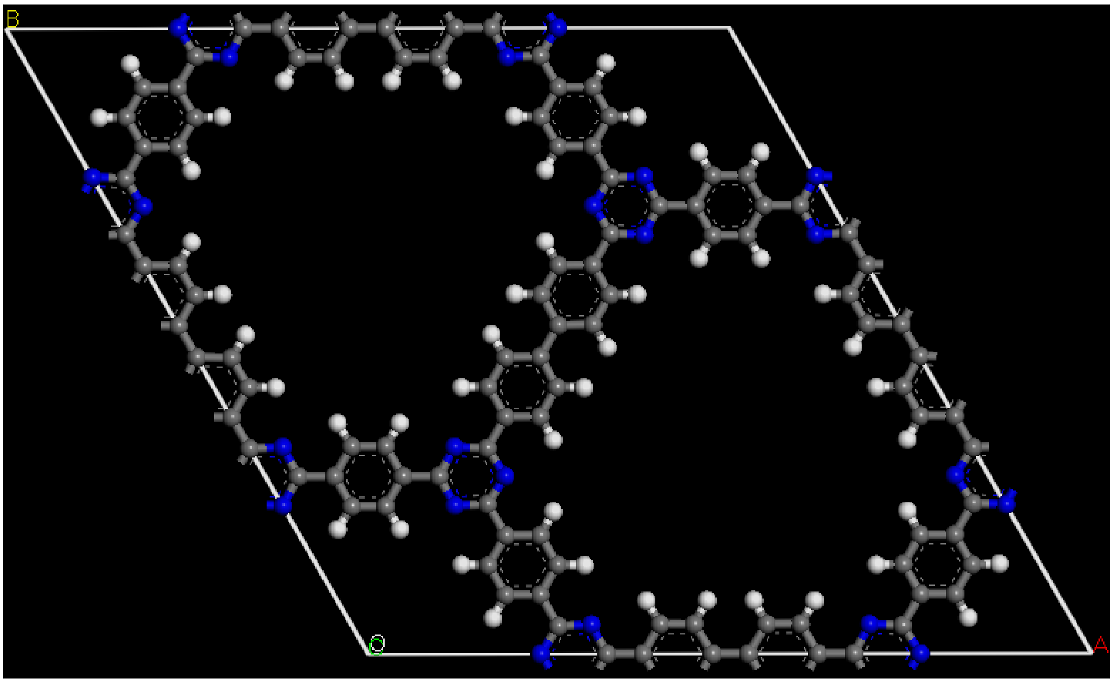

CTF-HUST-3

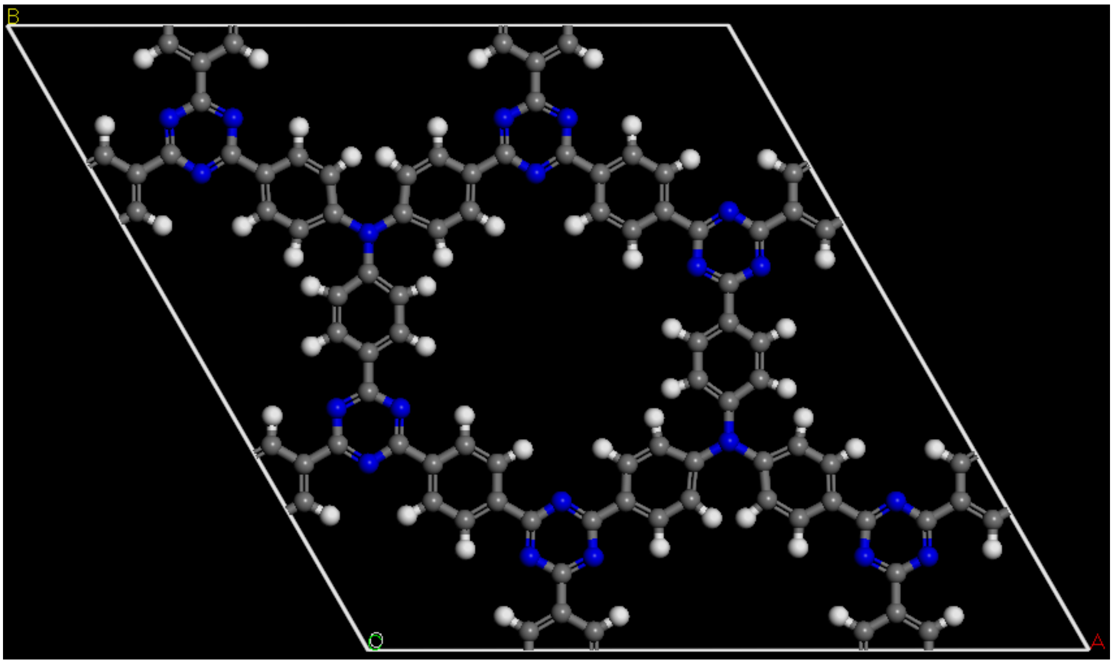

CTF-HUST-4

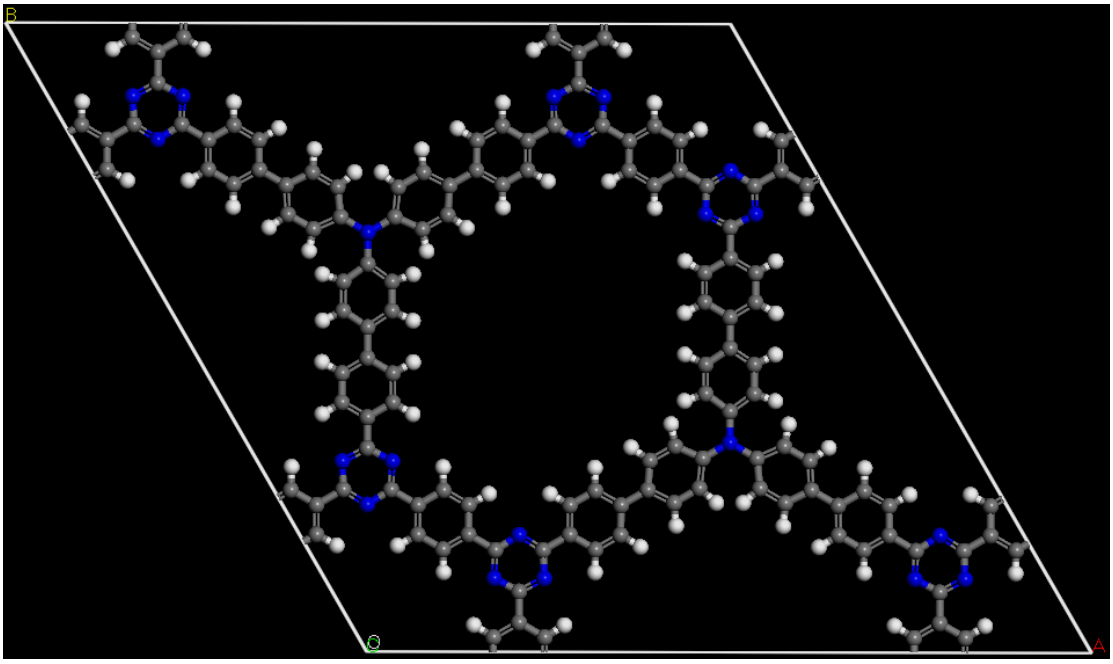

5. Supporting References:

1. S. Biswas, S. Batra, " One-Step Synthesis of 2-Amino-5*H*-pyrimido[5,4-b] indoles, Substituted 2-(1,3,5-triazin-2-yl)-1*H*-indoles, and 1,3,5-Triazines from Aldehydes", *Eur. J. Org. Chem.* 3492–3499 (2012).
2. Y. Shu, H. Lei, Y. N. Tan, M. Meng, X. C. Zhang, C. Y. Liu, " Tuning the electronic coupling in Mo_2–Mo_2 syntems by variation of the coordinating atoms of the bridging ligands", *Dalton Trans.*, **43**, 14756–14765 (2014).
3. Y. Zhao, T. Wang, L. Zhang, Y. Cui, B. Han, " Facile Approach to Preparing Microporous Organic Polymers through Benzoin Condensation", *ACS Appl. Mater. Interfaces*, **4**, 6975−6981 (2012).
4. D. Guo, R. Shibuya, C. Akiba, T. Kondo, J. Nakamura, *Science* **351**, 361-365 (2016).
5. G. Wang, J. Yang, J. Park, X. Gou, B. Wang, H. Liu, J. Yao, *J. Phys. Chem. C*, **112**, 8192-8195 (2008).
6. a) C. N. R. Rao, H. S. S. Matte Ramakrishna, U. Maitra, *Angew. Chem. Int. Ed.*, **52**, 13162–13185 (2013); b) X. Zhang, L. L. Hou, A. Ciesielski, P. Samorì, *Adv. Energy Mater.*, **6**, 1600671 (2016).
7. P. Hohenberg, W. Kohn, *Phys. Rev.*, **136**, B864 (1964).
8. G. Kresse, J. Hafner, *Phys. Rev. B*, **47**, 558 (1993).
9. P. E. Blochl, *Phys. Rev. B*, **50**, 17953 (1994).
10. G. Kresse, D. Joubert, *Phys. Rev. B*, **59**, 1758 (1999).
11. J. P. Perdew, J. A. Chevary, S. H. Vosko, K. A. Jackson, M. R. Pederson, D. J. Singh, C. Fiolhais, *Phys. Rev. B*, **46**, 6671 (1992).
12. J. P. Perdew, K. Burke, M. Ernzerhof, *Phys. Rev. Lett.*, **77**, 3865 (1996).
13. S. Grimme, J. Antony, S. Ehrlich, H. Krieg, *J. Chem. Phys.*, **132**, 154104 (2010).
14. P. Kuhn, M. Antonietti, A. Thomas, *Angew. Chem. Int. Ed.*, **47**, 3450–3453 (2008).
